# High frequency oscillations in relation to interictal spikes in predicting postsurgical seizure freedom

**DOI:** 10.1038/s41598-023-48764-4

**Published:** 2023-12-03

**Authors:** Jakob V. E. Gerstl, Alina Kiseleva, Lukas Imbach, Johannes Sarnthein, Tommaso Fedele

**Affiliations:** 1 University College London Medical School, London, UK; 2 Department of Neurosurgery, University Hospital and University of Zurich, Zurich, Switzerland; 3 Institute for Cognitive Neuroscience, HSE University, Myasnitskaya Ulitsa, 20, Moscow, Russian Federation 101000; 4 Swiss Epilepsy Center, Klinik Lengg, Zurich, Switzerland

**Keywords:** Neurological disorders, Epilepsy, Neurology

## Abstract

We evaluate whether interictal spikes, epileptiform HFOs and their co-occurrence (Spike + HFO) were included in the resection area with respect to seizure outcome. We also characterise the relationship between high frequency oscillations (HFOs) and propagating spikes. We analysed intracranial EEG of 20 patients that underwent resective epilepsy surgery. The co-occurrence of ripples and fast ripples was considered an HFO event; the co-occurrence of an interictal spike and HFO was considered a Spike + HFO event. HFO distribution and spike onset were compared in cases of spike propagation. Accuracy in predicting seizure outcome was 85% for HFO, 60% for Spikes, and 79% for Spike + HFO. Sensitivity was 57% for HFO, 71% for Spikes and 67% for Spikes + HFO. Specificity was 100% for HFO, 54% for Spikes and 85% for Spikes + HFO. In 2/2 patients with spike propagation, the spike onset included the HFO area. Combining interictal spikes with HFO had comparable accuracy to HFO. In patients with propagating spikes, HFO rate was maximal at the onset of spike propagation.

## Introduction

The global prevalence of epilepsy is over 70 million, making it one of the most common neurological conditions. About one third of these patients are affected by pharmacoresistant epilepsy, making them candidates for potentially curative resective surgery. However, 20–50% of the operated patients have recurrent seizures predominantly due to incomplete or inaccurate resections1. Epilepsy surgery primarily targets the complete resection of the epileptogenic zone (EZ), the brain area indispensable for the generation of new seizures^2^. In those patients undergoing intracranial EEG (iEEG) implantation for presurgical evaluation^3^, the seizure onset zone (SOZ) currently represents the gold standard to guide the identification of the putative EZ. The determination of the SOZ depends on the occurrence of seizures, which may be rare. The occurrence of spontaneous seizures during patients observation in an epilepsy monitoring unit might require prolonged iEEG recordings, which is associated with patient discomfort and potential complications^4,5^. Therefore, reliable biomarkers occurring in the interictal iEEG would be highly desirable.

Epileptiform potentials have served as the main interictal biomarker of reference for the EZ during preoperative assessment^6^. Intraoperative interictal spike frequency has been shown to drastically decrease in real time following the resection of epileptic foci^7^. Although these epileptic spikes confer a high sensitivity for identification of the true EZ, they are limited by low specificity. The low specificity of interictal spikes is likely due to their tendency to propagate to regions outside the EZ^8,9^. The resection of tissue presenting with spikes has, therefore, been associated with mixed outcomes^10^. High frequency oscillations (HFOs) have been proposed to be a more specific biomarker for the EZ because HFOs located outside the resected area are associated with poor surgical outcome^11–14^. The consideration of spikes and HFOs combined (Spike + HFO) has shown potential for more accurate identification of epileptogenic tissue in both invasive^15^ and non-invasive EEG^16^. Although an increasing number of studies have explored HFOs in relation to interictal spikes^17,18^, these events have previously not been considered in terms of spike propagation from the EZ.

This study aimed to test two hypotheses: firstly, whether Spike + HFO can improve EZ delineation compared to the individual constituents; secondly, whether HFOs can help identify crucial nodes in case of spike propagation. The clinical benefit of a biomarker is best evaluated in relation to postoperative seizure outcome. We here re-analyse a previously published dataset^12^, in which the HFO area, defined as the highest concentration of ripples co-occurring with fast ripples, provided 100% specificity, 57% sensitivity and 85% accuracy in the prediction of surgical outcome. We show that that (1) HFO were not significantly more accurate than interictal spikes in the prediction of postsurgical seizure freedom in our set of 20 patients; (2) the combination of Spike + HFO increased sensitivity but decreased specificity; (3) in the two patients with clear interictal spike propagation, the HFO area coincided with the spike onset.

## Results

**Table 1:** Summary of patient characteristics.

Patient	Age, Gender	Histology/pathology	Epilepsy	Electrode placement	Type of electrodes	Surgery	Nights	Intervals	HFO area resected?	Spike area resected	Spike + HFO area resected	Outcome (ILAE)	Postoperative follow-up (months)
1	25, M	HS and gliosis	TLE	MTL L, R	5 depth	sAHE; Les		28	Y	Y	Y	1	32
2	33, M	Glioma	TLE	MTL L, R	8 depth	sAHE; Les	2	13	Y	N	Y	1	30
3	20, F	HS	TLE	MTL L, R	5 depth	sAHE	5	39	Y	N	Y	1	28
4	20, F	HS	TLE	MTL L, R	8 depth	sAHE	6	34	Y	N	Y	1	42
5	40, M	HS	TLE	MTL L, R	8 depth	sAHE	5	35	Y	Y	Y	1	14
6	48, M	HS	TLE	MTL L, R	8 depth	sAHE	5	35	Y	Y	Y	1	12
7	25, M	HS	TLE	MTL L, R	8 depth	sAHE	1	1	Y	N	N	3	53
8	21, F	HS	TLE	MTL L, R	8 depth	sAHE	2	16	Y	Y	Y	3	28
9	52, M	HS	TLE	MTL L, R	8 depth	sAHE	2	12	Y	N	N	5	46
10	37, M	FCD 2b	ETE	Pr R	1 grid 8 × 4; 2 strips 4 × 1	Les	0	6	Y	Y	Y	1	48
11	36, M	FCD 2b	ETE	F R	1 grid 8 × 8; 1 depth	Les	3	19	Y	N	N	1	38
12	49, M	Ganglioglioma	ETE	T-Lat L	1 grid 8 × 4; 1 depth	Les	4	25	Y	Y	Y	1	37
13	17, M	FCD 1a	ETE	P R	1 grid 8 × 8; 1 depth	Les	2	16	Y	Y	Y	1	25
14	46, F	FCD 1b	ETE	P R	2 grids 8 × 2; 1 strip 6 × 1; 1 strip 4 × 1; 1 depth	Les	2	13	Y	N	Y	1	36
15	31, F	Gliosis	ETE	T-Lat L	1 grid 8 × 4; 2 strips 4 × 1	Les	4	28	Y	N	N	1	27
16	17, F	FCD 2a	ETE	F-Pr-C L	1 grid 8 × 4; 1 depth	Les	2	17	Y	Y	Y	1	28
17	30, M	FCD 2a	ETE	F R	2 grids 8 × 2; 1 depth	Les	1	1	N	Y	Y	5	46
18	40, M	FCD 2a	ETE	P R	2 strips 6 × 1; 1 depth	Les	1	6	N	N	N	5	38
19	38, M	Cavernoma	ETE	T P	1 grids 8 × 4; 1 grids 8 × 2	Les	2	13	N	N	N	6	18
20	17, M	FCD 3	ETE	O T	1 grids 8 × 2	Les	4	29	N	N	N	5	21

Abbreviations: *C* central, *depth* depth electrode, *ETE* extratemporal lobe epilepsy, *F* frontal, *FCD* focal cortical dysplasia, *FR* fast ripple, *FRandR* FR occurring together with ripple, *grid* grid electrode, *HFO* high frequency oscillation, *HS* hippocampus sclerosis, *ILAE* International League Against Epilepsy, *MTL* mesial temporal lobe, *L* left, *Lat* lateral, *Les* lesionectomy, *O* occipital, *P* parietal, *Pr* precentral, *R* right, *sAHE* selective amygdala-hippocampectomy, *strip* strip electrode, *T* temporal, *TLE* mesial temporal lobe epilepsy.

### Postsurgical seizure freedom and its prediction

Spikes and HFO were area were identified in all patients. Spikes + HFO area in 19/20 patients. The Spike area included a larger number of contacts of the HFO area (Wikoxon signed rank, *P* = 0.02). Of a total 20 patients (Table 1), 13 (65%) achieved seizure freedom (ILAE 1).

All these 13 patients had the HFO area resected, seven the Spike area resected and 11 the Spike + HFO area resected. Of the seven patients (35%) with recurrent seizures (ILAE 2–6), three had the HFO area resected, two had the spike area resected and two had the Spike + HFO area resected. The accuracy in the prediction of postsurgical seizure freedom was 85% for HFO, 60% for Spikes and 79% for Spike + HFO (Table 2). Prediction power was similar between HFO and Spikes + HFO, but not significantly higher than in Spikes (z-test, *P* > 0.05).

**Table 2:** Biomarker (spikes, HFO, spikes + HFO) and seizure outcome.

	HFO	Spikes	Spikes + HFO
Sensitivity [%] TP/(TP + FN)	57 [18 90]	71 [29 96]	67 [22 96]
Specificity [%] TN/(TN + FP)	100 [75 100]	54 [25 81]	85 [55 98]
PPV [%] TP/(TP + FP)	100 [40 100]	45 [17 77]	67 [22 96]
NPV [%] TN/(TN + FN)	81 [54 96]	78 [40 97]	85 [55 98]
Accuracy [%] (TP + TN)/N	85 [62 97]	60 [36 81]	79 [54 94]

Values in brackets denote the 95% confidence interval. HFO are defined as fast ripples co-occurring with ripples. Abbreviations: *FN* False Negative, *FP* False Positive, *NPV* Negative Predictive Value, *PPV* Positive Predictive Value, *TN* True Negative, *TP* True Positive, *N* number of patients.

The specificity in predicting seizure outcome was 100% for HFO, 54% for Spikes and 85% for Spike + HFO. Sensitivity was 57% for HFO, 71% for Spikes and 67% for Spike + HFO.

Six patients showed disagreement between HFO and Spike + HFO. In two patients (patient 7 and 9) with poor surgical outcome and mesial temporal lobe hippocampal sclerosis, prediction with Spike + HFO improved on HFO alone. In both patients the HFO area was resected and the remaining contralateral Spike + HFO area remained unresected. In four patients (patients 11, 15, 17 and 18), three of which had lesional extratemporal lobe epilepsy. Patient 11 and 15 achieved seizure freedom and had the HFO area resected with the Spike area not resected and Spike + HFO area partially resected. Both patients 17 and 18 had seizure recurrence. In patient 17, the HFO area was not resected, the Spike area and the Spike + HFO area were completely resected. Finally, in patient 18, the HFO focus was not resected, the Spike area was not resected and the Spike + HFO area could not be clearly defined due to the absence of co-occurring spikes and HFO events. In this patient, both HFO and Spikes were indicative of the poor outcome.

### HFO and spike propagation

**Figure 1:**
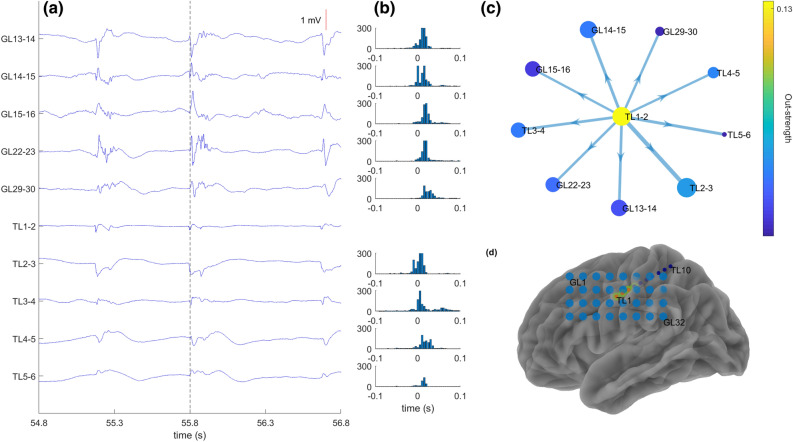
Spikes propagate from the HFO area in patient 16. (**a**) Signal from representative bipolar channels demonstrating the propagation pattern from the leading channel TL1-2. Spike latency in the leading channel is indicated by a vertical line. (**b**) Post-stimulus time histogram for all receiving channels with spike count as a function of latency after the leading channel. (**c**) Propagation pattern in the directed graph. A node (circle) indicates an electrode. The diameter of the nodes denotes the spike rate (Spike/minute). The colour of the node denotes its out-strength (sum of the conditional probabilities of this node to lead other nodes). Each arrow (arrow) marks the direction from the leading node to the receiving node. The width of the arrow denotes its weight in the adjacency matrix (**d**) Implantation scheme with grid electrode contacts in light blue and depth electrode contracts in dark blue. Electrodes with high spike rate and high HFO rate are marked in orange. The resected area is highlighted in green. The patient achieved seizure freedom.

We observed clear spike propagation patterns in 2 patients (16, 20). Patient 16 (ILAE 1—Fig. 1) was implanted with one depth electrode recording in proximity of a FCD lesion and one grid electrode was placed over the left sensorimotor cortex. Spike rates showed clear peaks at the tip of the depth electrode and in the centre of the grid electrode. Spikes detected at the tip of the depth electrode propagated towards the overlying grid. The HFO area included the source of the spike propagation pattern. The resection of the Spike + HFO area was associated with seizure freedom.

**Figure 2:**
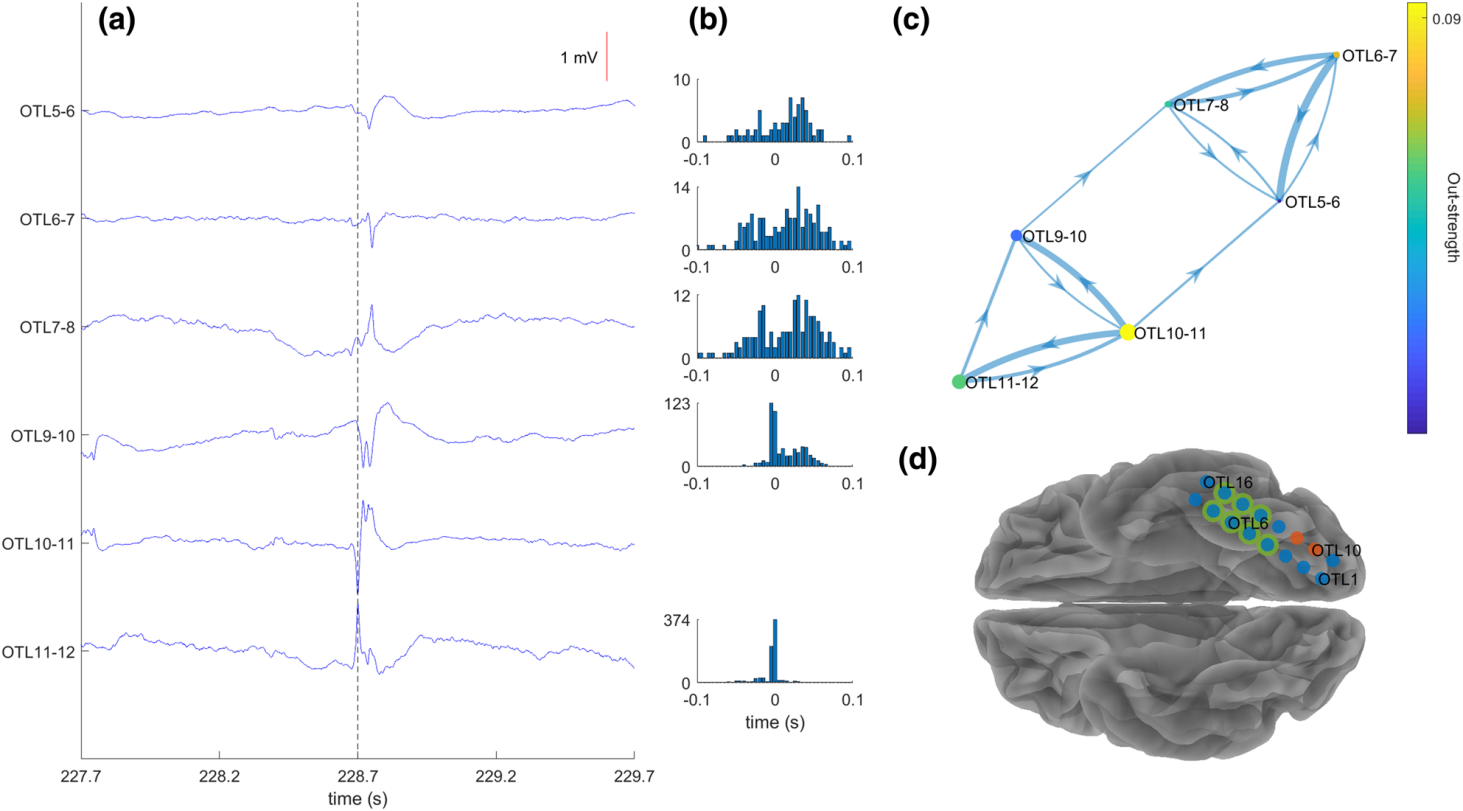
Spikes propagate from the HFO area in patient 20. (**a**) Signal from representative bipolar channels demonstrating the propagation pattern from the leading channel OTL10-11. Spike latency in the leading channel is indicated by a vertical line. (**b**) Post-stimulus time histogram for all receiving channels with spike count as a function of latency after the leading channel. (**c**) Propagation pattern in the directed graph. A node (circle) indicates an electrode. The diameter of the nodes denotes the spikes rate (events/minute). The colour of the node denotes its out-strength (sum of the conditional probabilities of this node to lead other nodes). Each arrow (arrow) marks the direction from the leading node to the receiving node. The width of the arrow denotes its weight in the adjacency matrix (**d**) Implantation scheme with grid electrode contacts in light blue. Electrodes with high spike rate and high HFO rate are marked in orange. The resected area is highlighted in green and does not include the HFO area. The patient suffered from recurrent seizures.

Patient 20 (ILAE 5—Fig. 2) was implanted with a grid on the left basal occipitotemporal cortex. The Spike + HFO area was also the origin of spike propagation (OTL10-11). The patient underwent a lesionectomy for FCD type III and had contacts OTL4-7 and OTL13-15 resected. However, spike propagation, HFOs and the Spike + HFO area all pointed towards unresected tissue, contacts OTL10 and OTL11, and the patient subsequently suffered from postoperative seizure recurrence.

## Discussion

The present study compared the predictive value of HFOs, spikes and Spike + HFO as biomarkers for epilepsy surgery. Consistent with previous literature, a higher specificity was found for HFOs than for interictal spikes, whereas the sensitivity was higher for interictal spikes^11,19,20^. Moreover, PPV, NPV and accuracy were all higher for HFOs. The combination of spikes and HFOs increased sensitivity over HFOs whereas there was a trade-off in specificity. These results confirm early reports which correlated the volume of resected HFO area with seizure freedom on a population level^21,22^ as summarised in a later meta-analysis^23^. These early studies were, however, conducted on a population level precluding conclusions in the individual patient and did not evaluate the accuracy of different biomarkers. The fact that HFOs do indeed confer a high specificity on the patient level was shown using the same dataset as the present study^12^ and has been confirmed in independent datasets^24–26^. Interestingly, a subsequent study revealed comparable performance between spikes and HFOs, and that the combination of spikes and HFO provided the best estimation of the EZ^15^. There are however limitations to HFO analysis such as sleep variation and the presence of physiological HFOs, and the authors recommend continued developments of atlases of physiological HFOs^27^.

In our study, both patients in which Spike + HFO improved on HFOs had mesial temporal lobe hippocampal sclerosis. In both patients, Spikes also made the correct prediction, the combination of HFOs and Spikes therefore brought little utility in this cohort. This contrasts with the previous report where Spike + HFO improved on both HFOs and Spikes alone^15^. Moreover, Roehri et al. estimated combined interictal spike and HFO areas by multiplying the channel spike and HFO rates. Using intracranial hybrid micro and macroelectrodes, Guth et al. demonstrated that single units significantly increased their firing rate during the combined spike and HFO component but did not alter their firing during spikes devoid of HFO^17^. Resection of such areas has been associated with favourable outcome^28^.

In our cohort, two patients with clear spike propagation were identified. In both patients, the estimated EZ coincided with the HFO area, correctly predicting seizure outcome. Interictal spike propagation is a well-known phenomenon which has been demonstrated in previous early studies^29^. More recent contributions have highlighted the importance of delineating the spike onset and propagation path^30,31^ and that the resection of the source of spike propagation is associated with good outcome^32^. During real time intraoperative monitoring of spikes in anterior temporal lobe resections, a gradual decrease of spikes was reported following superior temporal gyrus resection with continued decrease following en bloc removal of the lateral temporal pole^7^. Moreover, this immediate decrease in spikes was observed in ipsilateral and contralateral destinations of spike propagation from the resected epileptic foci. Those patients whose intraoperative real time decrease in spikes could be monitored were seizure free at one year follow-up. Furthermore, resection of interictal spike onset zones has been associated with favourable seizure outcomes in a recent paediatric series^31^. Although sources of spike propagation tend to be associated with epileptogenic tissue, the complexity of the propagation pathway does not only mimic the spread of ictal discharges but can also travel in opposite directions^9^. The consideration of pathological HFO areas with co-occurring spikes as generators within spike propagation networks as demonstrated in the present study therefore adds further value to these signatures of epileptogenicity. The ability to intraoperatively monitor these HFO generators in real time would moreover be of value and is underway^33^. Further, larger series to confirm HFO areas more clearly as biomarkers of the EZ are required^34^.

Some design choices in our methods of analysis merit further discussion. First*,* when we analyse events of interest and label them as either epileptiform HFO or not, the labelling may contain some uncertainty. We might either miss epileptiform HFO or we might erroneously count artefacts or physiological HFO in the HFO rate. To rule out spurious artefacts, we require temporal consistency of the HFO rates^12,13^. To rule out physiological HFO in the MTL, patients have performed cognitive tasks that activated their MTL but did not modulate the rate of epileptiform HFO^35^. Further, the rates of physiological HFO do not sensitively affect the spatial profile of epileptiform HFOs^35^. Second, our statistical threshold to define biomarker areas (95^th^ percentile over Spike, HFO and Spike + HFO rates) keeps the number of contacts in the area small. The number is even reduced by the requirement that the HFO rate must be consistently high over time. The 95th percentile refers to the rates of the channels composing the montage. This does not correspond to the 5% of the channels with the highest rates. As such, the number of channels can vary, according to the distribution tail. This thresholding strategy can penalize patients with a large EZ, since identifies a limited volume characterized by high biomarker rates. However, the resection area was limited to a small number of contacts. We observe a statistical higher number of contacts populating the Spike area than the HFO area. In our previous studies, the number of bipolar channels in the HFO area was ≤ 5 for all patients^12,24^. These numbers compare well with the number of channels in the SOZ which were in the same range. The small numbers reflect that in our centre only patients with focal epilepsy are selected for iEEG implantation and the spatial sampling with iEEG electrodes is low. Third, spikes were manually marked by experienced reviewers in consensus to improve on the interrater reliability^36,37^. Fourth, spike propagation in MTL might have been under detected given our limited spatial sampling in comparison with recent studies^38^. Finally, the size of cohort of 20 patients prevents broad generalization our findings. Still, we show examples where spikes propagation from the HFO area is in agreement with seizure outcome. Our findings should be verified in larger clinical populations^25^, which may be provided by multicentre cohorts^34^. In such settings, automatic interictal spike^39^ and HFO detection may be deployed to standardize the definition of interictal biomarkers across clinical centres.

Interictal biomarkers validated on clinical data support the delineation of the epileptic network. In this context, spikes, HFO and their consideration in a static and dynamic framework provides useful clinical information. In this study we showed that HFO were not more accurate than interictal spikes in prediction of postsurgical seizure freedom (z-test, P > 0.05). Combining spikes with HFO increased sensitivity but decreased specificity. Moreover, the observed interictal spike propagation onset corresponded with the HFO area.

## Methods

### Patient selection

Consecutive patients from March 2012 to April 2016 with pharmacoresistant focal epilepsy were included if they underwent intracranial EEG recordings with either stereoelectroencephalography (sEEG), subdural recordings or both (Table 1). Only patients who consequently underwent resective surgery with > 12 month follow up were included. Seizure freedom was assessed at follow-up visits using the International League Against Epilepsy (ILAE) classification.

### Ethics statement

Retrospective collection and analysis of patient data was approved upfront by the local research ethics committee (Kantonale Ethikkommission KEK-ZH-Nr. PB-2016-02055) and patients gave written informed consent. The study was conducted in agreement with all relevant guidelines and regulations.

### Electrode types, implantation sites and resective surgical planning

Findings of non-invasive presurgical evaluation determined the placement of sEEG and subdural grids and strips. In temporal lobe epilepsy (TLE) patients, sEEG depth electrodes (1.3 mm diameter, 8 contacts of 1.6 mm length, spacing between contacts centres 5 mm, ADTech^®^, www.adtechmedical.com) were stereotactically implanted into the amygdala, entorhinal and perirhinal cortices and hippocampus bilaterally. In extratemporal epilepsy patients (ETE) patients, a combination of sEEG and subdural grid and strip electrodes (contact diameter 4 mm with a 2.3 mm exposure, spacing between contact centres 10 mm, ADTech^®^) were used. Resective surgery was planned according to non-invasive and intracranial investigations without the use of HFO analysis.

### Data acquisition

Intracranial EEG (iEEG) was recorded by an ATLAS recording system (0.5–1000 Hz pass-band, Neuralynx, www.neuralynx.com) at 4000 Hz sampling frequency and down-sampled to 2000 Hz for HFO analysis. Scalp EEG according to the 10–20 system, with minor adjustments for surgical scalp lesions, and submental electromyogram (EMG) were recorded. iEEG was recorded against a common intracranial reference and subsequently transformed to bipolar channels for further analysis.

### Data selection

We focussed on interictal slow-wave sleep that promotes low muscle activity and high HFO rates. Sleep scoring was based on scalp EEG, electro-oculogram, EMG and video recordings.

To rule out the influence of seizure activity, the analyzed EEG recordings were at least three hours apart from ictal events. These selection criteria resulted in up to six intervals of five minutes per night in some patients. The number of nights and intervals varied across the patient group (Table 1). Electrodes that were placed close to motor or language areas were tested with tasks that elicited motor or language ERPs as a standard clinical procedure to identify the contacts covering the eloquent cortex. All contacts evoking motor or language responses were excluded. In TLE patients, the three most mesial bipolar channels were included, while lateral contacts were excluded from the analysis, as they were not relevant for the surgical hypothesis.

### Prospective definition of HFOs and visual interictal spike detection

We define as an epileptiform HFO the co-occurrence of a ripple and a fast ripple, which we denote by the term HFO throughout the paper, consistent with our prior work on this and other independent datasets^12,24,26^. HFOs were defined prospectively using an automated detector developed by our group which had previously been trained, tested and validated to detect visually marked events in datasets from the Montreal Neurological Institute^19^. The HFO detection was conducted separately for ripples (band-pass 80–240 Hz, stopband 70 Hz and 250 Hz, FIR equiripple filter with stopband attenuation 60 dB) and FRs (band-pass 250–490 Hz, stopband 240 Hz and 500 Hz). The code is freely available at the HFO detectors repository on github (https://github.com/HFO-detect/ HFO-detect-matlab). The iEEG data with the markings of our HFO events is freely available at CRCNS.org http://dx.doi.org/10.6080/K06Q1VD5. Links to further publications on the detector can be found on https://hfozuri.ch.

Interictal epileptic spikes were visually marked over the full dataset by two reviewers (J.V.E.G. and L.I.). The criterion for interictal spike detection was an isolated triangular wave clearly distinct from the background^40^. Conflicts were resolved by consensus. Interictal spikes were marked using a cursor, marked latencies were subsequently automatically adjusted to fit the spike peak. Combined Spikes and HFO events were defined as HFO occurring + /− 50 ms around the identified spike peak. The interface was realized by custom MATLAB scripts.

### Spike propagation

To determine the spike onset in case of spike propagation, we employed a community detection algorithm which generated propagation graphs with each node representing a bipolar channel^41^. A weighted adjacency matrix was initially created, where each element contained the conditional probability of paired propagation from site to site. More specifically, every column of the matrix represented the out-strength (the probability of each electrode to lead every other electrode) and each row contained the in-strength (the probability of each electrode to follow every other electrode). Next, a spatial model was built, considering site proximity, and extracting propagation clusters according to modularity statistics, since all electrodes inside a given cluster have strong local connections and have weaker connections with electrodes outside the cluster. A permutation test was used to evaluate the statistical significance of the modularity of each cluster.

In the propagation graphs of each cluster, nodes of the graph corresponded to electrodes inside the cluster. The size of the nodes corresponded to spike rate (events/minute), while the colour of the electrode corresponded to out-strength. The edges and their weights were given by the adjacency matrix, where the direction of arrows went from the leading electrode to the following electrode. For the clearer visualisation, the nodes had thresholds half of the mean of spike rates and edges at a quarter of the maximum value of the adjacency matrix.

### Definition of spike, HFO and spike + HFO areas by rate thresholding

**Figure 3:**
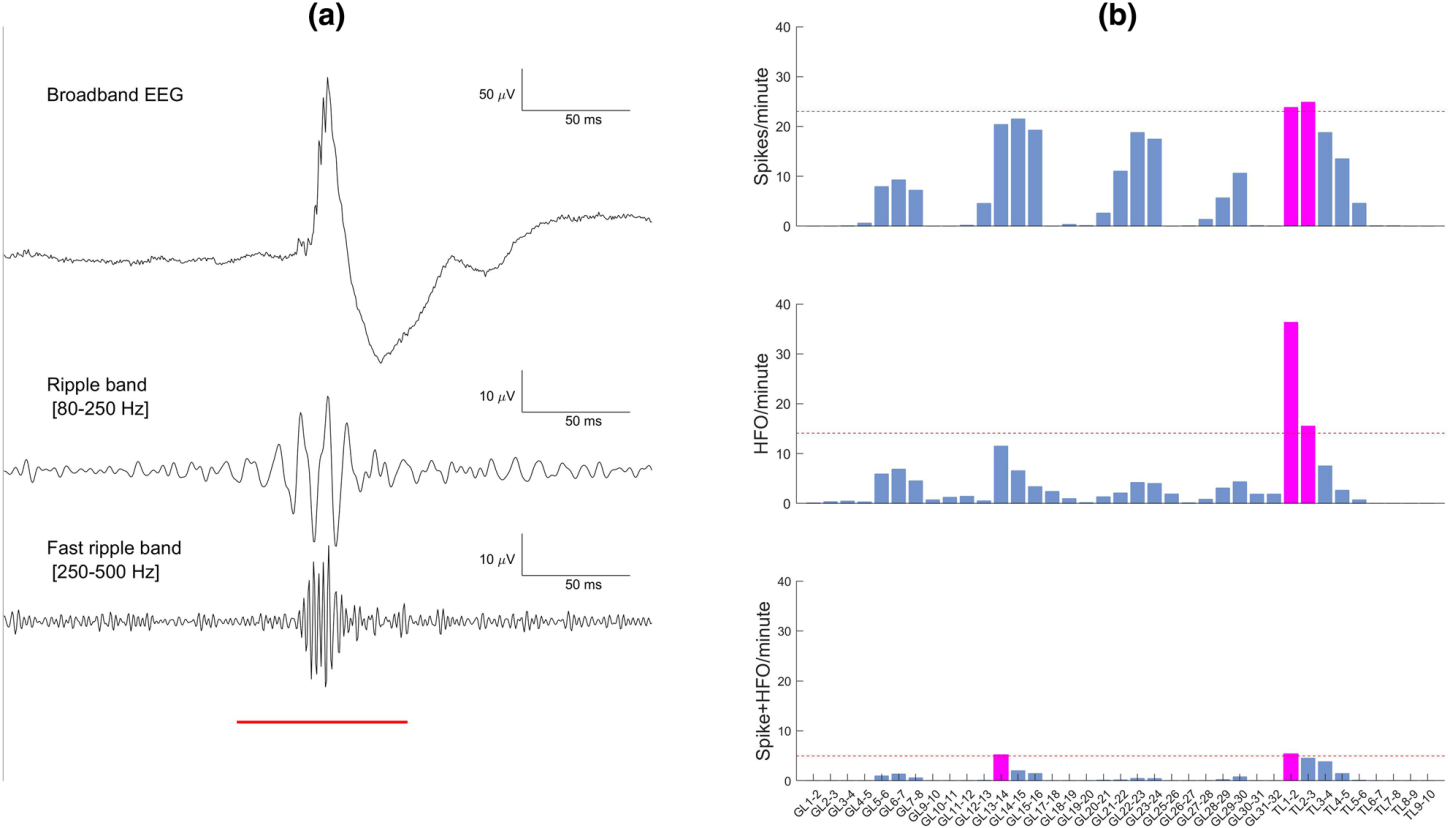
Biomarker and biomarker area. (**a**) Example of an event where a Spike (top) occurred simultaneously with an HFO, defined as the concurrence of a ripple (80–250 Hz) and a FR (250–500 Hz). This event was classified as Spike, HFO and Spike + HFO. The event duration is marked by the red horizontal line. (**b**) Rate (event/minute) for the Spike, HFO and Spike + HFO for each bipolar channel. Magenta channels have rate above threshold (horizontal line) and define the Spike area, the HFO area, and the Spike + HFO area.

We defined a biomarker area as the ensemble of channels where the rate of the event of interest exceeded the 95th percentile threshold of the spatial distribution of event rate. This definition led to obtain the Spike area, the HFO area, and the Spike + HFO area in each patient. An example of a Spike + HFO event is show in Fig. 3A. An example biomarker area definition is provided in Fig. 3B. This thresholding strategy ensures capturing the main focus of the biomarker area.

### Clinical validation of spikes and HFOs against seizure outcome

**Figure 4:**
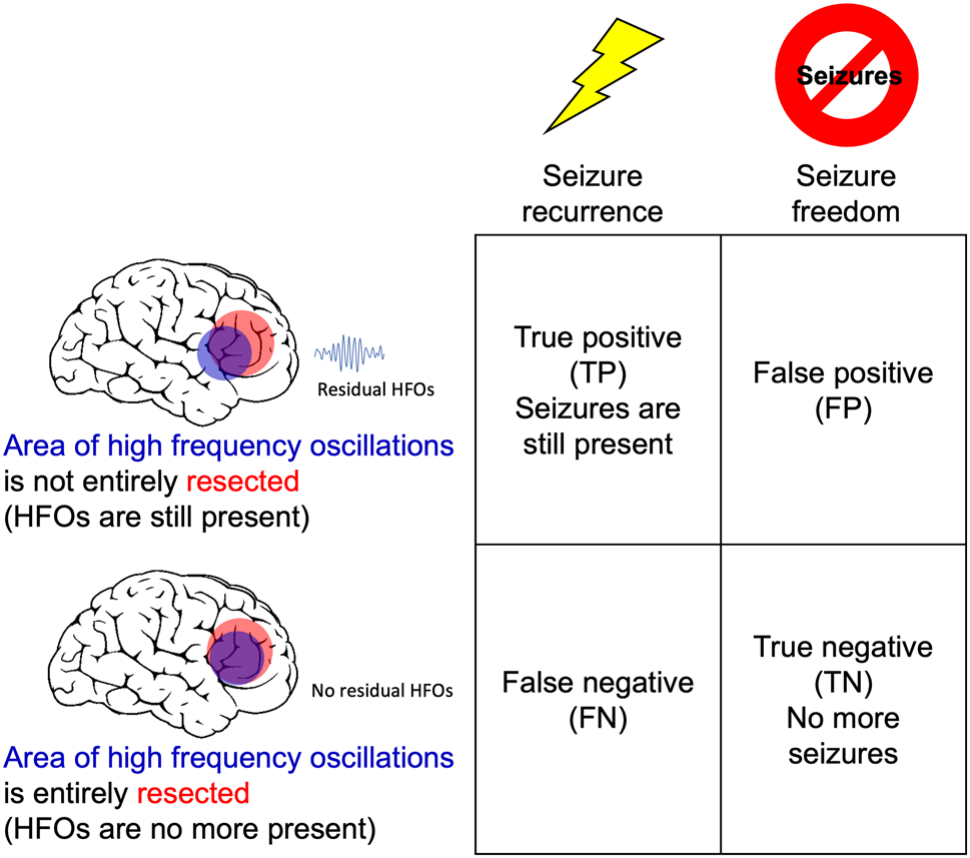
Confusion matrix for outcome prediction. We here illustrate the prediction of the HFO area. The analogous matrix holds for the Spike area and the Spike + HFO area.

We evaluated whether HFO, Spike and Spike + HFO were included in the resection area to quantify their predictive values with respect to postsurgical seizure outcome^12,24,26,42^. The primary outcome is seizure freedom (ILAE 1). Considering HFOs, we define as true positive (TP) a patient where the HFO area is not fully located within the resected area (RA), i.e. at least one channel of the HFO area is not resected and the patient suffers from recurrent seizures (ILAE 2–6). We define as false positive (FP), a patient where the HFO area is not fully located inside the RA but who achieves seizure freedom (ILAE 1). We define as false negative (FN), a patient where the HFO area is fully located within the RA but who suffers from recurrent seizures. We define as true negative (TN), a patient where the HFO area is fully located inside the RA and who becomes seizure-free. We use these values as the elements of the confusion matrix (Fig. 4). The positive-predictive value is calculated as PPV = TP/(TP + FP), negative-predictive value as NPV = TN/(TN + FN), sensitivity = TP/(TP + FN), specificity = TN/(TN + FP) and accuracy = (TP + TN)/N. The same holds for Spike and Spike + HFO areas. We emphasize that these definitions allow us to compare spikes and HFO in terms of sensitivity and specificity. Here sensitivity measures the ability to correctly identify incomplete resections while specificity measures the ability to correctly identify complete resections. We compared the accuracy of different biomarker through hypothesis test^43^, with significance at *P*-value = 0.05 (Supplementary Information).

### Supplementary Information

Supplementary Tables.
