# Supplementary material for: High frequency oscillations in relation to interictal spikes in predicting postsurgical seizure freedom
Source: Sci Rep. 2023 Dec 3;13:21313. doi: 10.1038/s41598-023-48764-4 (PMC10693609; doi:10.1038/s41598-023-48764-4)
Supplement: Supplementary file 1 — Supplementary Tables. [file 41598_2023_48764_MOESM1_ESM.docx]

**Supplementary Material**

| **Patient 1 (ILAE 1)** | **Channels** | **Ripples** | **FR** | **FRandR** | **Spikes** | **S + HFO** |
| --- | --- | --- | --- | --- | --- | --- |
| **ILAE 1** | **'IAR1-2'** | 33.84 | 2.95 | 0.26 | 0.11 | 0.01 |
|  | **'IAR2-3'** | 55.20 | 3.16 | 0.57 | 0.20 | 0.02 |
|  | **'IAR3-4'** | 40.18 | 2.88 | 0.49 | 0.24 | 0.03 |
|  | **'IAR4-5'** | 26.68 | 2.80 | 0.20 | 0.72 | 0.06 |
|  | **'IAR5-6'** | 31.14 | 2.84 | 0.35 | 0.80 | 0.06 |
|  | **'IPR1-2'** | 0.62 | 2.44 | 0.00 | 0.01 | 0.00 |
|  | **'IPR2-3'** | 1.68 | 2.57 | 0.01 | 0.28 | 0.00 |
|  | **'IPR3-4'** | 11.77 | 2.41 | 0.04 | 0.26 | 0.01 |
|  | **'AHR1-2'** | 19.81 | 3.33 | 0.91 | 4.62 | 0.70 |
|  | **'AHR2-3'** | 21.40 | 4.76 | 2.39 | 4.83 | 1.47 |
|  | **'AHR3-4'** | 19.72 | 2.98 | 0.40 | 3.33 | 0.11 |
|  | **'AL1-2'** | 3.21 | 2.34 | 0.03 | 0.03 | 0.00 |
|  | **'AL2-3'** | 0.61 | 2.68 | 0.00 | 0.03 | 0.00 |
|  | **'AL3-4'** | 0.24 | 2.62 | 0.01 | 0.00 | 0.00 |
|  | **'AR1-2'** | 47.77 | 5.26 | 2.68 | 5.06 | 0.77 |
|  | **'AR2-3'** | 48.24 | 5.10 | 2.67 | 5.19 | 0.77 |
|  | **'AR3-4'** | 4.31 | 2.82 | 0.09 | 0.94 | 0.01 |
|  | **'HL1-2'** | 49.27 | 2.55 | 0.18 | 0.01 | 0.00 |
|  | **'HL2-3'** | 60.56 | 2.20 | 0.20 | 0.04 | 0.00 |
|  | **'HL3-4'** | 105.09 | 2.48 | 0.37 | 0.04 | 0.00 |
|  | **'PHR1-2'** | 22.78 | 10.23 | 6.70 | 11.27 | 5.10 |
|  | **'PHR2-3'** | 20.17 | 4.86 | 2.37 | 12.05 | 2.03 |
|  | **'PHR3-4'** | 11.82 | 2.95 | 0.36 | 10.09 | 0.26 |
| Threshold |  |  |  |  | **11.54** | **3.10** |

| **Patient 2** | **Channels** | **Ripples** | **FR** | **FRandR** | **Spikes** | **S + HFO** |
| --- | --- | --- | --- | --- | --- | --- |
| **ILAE 1** | **'AL1-2'** | 44.3 | 9.1 | 4.6 | 3.1 | 0.9077 |
|  | **'AL2-3'** | 17.7 | 4.2 | 1.4 | 3.3 | 1.2769 |
|  | **'AL3-4'** | 5.1 | 3.4 | 0.9 | 2.7 | 0.8462 |
|  | **'AR1-2'** | 52.6 | 16.3 | 7.5 | 1.7 | 0.2769 |
|  | **'AR2-3'** | 16.2 | 3.5 | 1.0 | 2.0 | 0.6923 |
|  | **'AR3-4'** | 1.4 | 1.6 | 0.0 | 1.2 | 0.0308 |
|  | **'EL1-2'** | 2.6 | 2.0 | 0.0 | 0.0 | 0 |
|  | **'EL2-3'** | 1.5 | 2.4 | 0.0 | 0.0 | 0 |
|  | **'EL3-4'** | 2.7 | 1.8 | 0.1 | 0.2 | 0 |
|  | **'ER1-2'** | 51.4 | 5.4 | 2.7 | 0.0 | 0 |
|  | **'ER2-3'** | 11.7 | 2.6 | 0.2 | 0.0 | 0 |
|  | **'ER3-4'** | 0.6 | 1.7 | 0.0 | 0.1 | 0.0154 |
|  | **'HL1-2'** | 4.8 | 2.7 | 0.8 | 0.5 | 0.2308 |
|  | **'HL2-3'** | 1.8 | 1.9 | 0.0 | 0.5 | 0 |
|  | **'HL3-4'** | 1.8 | 2.2 | 0.0 | 0.5 | 0 |
|  | **'HR1-2'** | 71.4 | 14.1 | 6.7 | 0.7 | 0.3077 |
|  | **'HR2-3'** | 19.7 | 2.6 | 0.3 | 0.7 | 0.1385 |
|  | **'HR3-4'** | 1.6 | 2.6 | 0.0 | 0.6 | 0.0308 |
|  | **'PL1-2'** | 16.3 | 13.1 | 1.7 | 1.6 | 0.9077 |
|  | **'PL2-3'** | 2.8 | 6.0 | 0.2 | 1.6 | 0.1077 |
|  | **'PL3-4'** | 0.4 | 2.5 | 0.1 | 0.9 | 0 |
|  | **'PR1-2'** | 48.9 | 16.2 | 9.8 | 2.58461538 | 0.9846 |
|  | **'PR2-3'** | 18.1 | 6.9 | 3.7 | 2.6 | 1.6923 |
|  | **'PR3-4'** | 0.8 | 2.5 | 0.0 | 1.2 | 0.0308 |
| Threshold |  |  |  |  | **3.17** | **1.4** |

| **Patient 3** | **Channels** | **Ripples** | **FR** | **FRandR** | **Spikes2** | **S2 + HFO** |
| --- | --- | --- | --- | --- | --- | --- |
| **ILAE 1** | **'AHL1-2'** | 20.2 | 15.5 | 9.1 | 2.45641026 | 2.3692 |
|  | **'AHL2-3'** | 7.0 | 7.3 | 2.6 | 2.47692308 | 0.8769 |
|  | **'AHL3-4'** | 2.1 | 2.5 | 0.0 | 0.32307692 | 0 |
|  | **'AHR1-2'** | 211.9 | 10.1 | 5.2 | 3.21538462 | 0.8769 |
|  | **'AHR2-3'** | 159.0 | 5.8 | 2.4 | 3.47179487 | 0.8564 |
|  | **'AHR3-4'** | 233.8 | 5.1 | 2.5 | 2.62564103 | 0.5333 |
|  | **'AL1-2'** | 1.6 | 2.4 | 0.0 | 0 | 0 |
|  | **'AL2-3'** | 0.7 | 2.3 | 0.0 | 0 | 0 |
|  | **'AL3-4'** | 0.2 | 2.5 | 0.0 | 0.00512821 | 0 |
|  | **'ECL1-2'** | 2.0 | 2.7 | 0.0 | 0 | 0 |
|  | **'ECL2-3'** | 0.4 | 2.7 | 0.0 | 0 | 0 |
|  | **'ECL3-4'** | 0.1 | 2.8 | 0.0 | 0.00512821 | 0 |
|  | **'PHL1-2'** | 8.0 | 3.5 | 1.2 | 0 | 0 |
|  | **'PHL2-3'** | 1.0 | 3.0 | 0.4 | 0 | 0 |
|  | **'PHL3-4'** | 0.7 | 6.8 | 0.1 | 0 | 0 |
| Threshold |  |  |  |  | 3.408 | 1.9962 |

| **Patient 4** | **Channels** | **Ripples** | **FR** | **FRandR** | **Spikes** | **S + HFO** |
| --- | --- | --- | --- | --- | --- | --- |
| **ILAE 1** | **'AL1-2'** | 1.0 | 2.3 | 0.0 | 0.0 | 0 |
|  | **'AL2-3'** | 0.9 | 2.3 | 0.0 | 0.0 | 0 |
|  | **'AL3-4'** | 0.2 | 2.8 | 0.0 | 0.1 | 0 |
|  | **'AR1-2'** | 39.6 | 23.4 | 14.0 | 0.1 | 0.0529 |
|  | **'AR2-3'** | 32.5 | 18.0 | 12.3 | 0.1 | 0.0529 |
|  | **'AR3-4'** | 11.0 | 5.5 | 2.3 | 0.2 | 0.0059 |
|  | **'EL1-2'** | 0.3 | 2.8 | 0.0 | 0.0 | 0 |
|  | **'EL2-3'** | 0.1 | 2.9 | 0.0 | 0.0 | 0 |
|  | **'EL3-4'** | 0.1 | 2.4 | 0.0 | 0.2 | 0 |
|  | **'ER1-2'** | 3.9 | 2.9 | 0.0 | 0.0 | 0 |
|  | **'ER2-3'** | 1.7 | 2.9 | 0.0 | 0.0 | 0 |
|  | **'ER3-4'** | 3.0 | 2.5 | 0.0 | 0.0 | 0 |
|  | **'HL1-2'** | 140.6 | 5.7 | 1.7 | 5.2 | 0.7294 |
|  | **'HL2-3'** | 128.2 | 5.3 | 1.6 | 5.3 | 0.7824 |
|  | **'HL3-4'** | 63.8 | 4.2 | 1.7 | 0.6 | 0.1529 |
|  | **'HR1-2'** | 41.6 | 19.0 | 9.0 | 2.6 | 1.9471 |
|  | **'HR2-3'** | 36.5 | 19.0 | 6.4 | 3.3 | 1.4176 |
|  | **'HR3-4'** | 20.4 | 13.5 | 5.0 | 3.5 | 1.3 |
|  | **'PL1-2'** | 82.6 | 2.5 | 0.6 | 0.0 | 0 |
|  | **'PL2-3'** | 160.3 | 2.9 | 1.2 | 0.0 | 0 |
|  | **'PL3-4'** | 44.6 | 2.4 | 0.2 | 0.2 | 0 |
|  | **'PR1-2'** | 12.2 | 8.4 | 5.2 | 0.4 | 0.4706 |
|  | **'PR2-3'** | 3.7 | 3.7 | 0.6 | 0.4 | 0.1176 |
|  | **'PR3-4'** | 0.6 | 2.7 | 0.0 | 0.0 | 0 |
| Threshold |  |  |  |  | 5.25 | 1.577 |

| **Patient 5** | **Channels** | **Ripples** | **FR** | **FRandR** | **Spikes** | **S + HFO** |
| --- | --- | --- | --- | --- | --- | --- |
| **ILAE 1** | 'AL1-2' | 14.2 | 3.8 | 0.7 | 1.6 | 0.1714 |
|  | 'AL2-3' | 3.3 | 2.8 | 0.3 | 1.6 | 0.2286 |
|  | 'AL3-4' | 1.8 | 2.4 | 0.0 | 0.1 | 0 |
|  | 'AR1-2' | 43.0 | 12.6 | 6.2 | 1.2 | 0.3029 |
|  | 'AR2-3' | 30.1 | 2.6 | 0.3 | 1.2 | 0.0914 |
|  | 'AR3-4' | 8.2 | 2.4 | 0.1 | 0.5 | 0.0229 |
|  | 'EL1-2' | 2.8 | 2.6 | 0.3 | 0.0 | 0 |
|  | 'EL2-3' | 2.6 | 2.4 | 0.1 | 0.0 | 0 |
|  | 'EL3-4' | 1.1 | 2.1 | 0.0 | 0.2 | 0 |
|  | 'ER1-2' | 6.9 | 3.2 | 0.4 | 0.0 | 0.0057 |
|  | 'ER2-3' | 4.7 | 2.5 | 0.1 | 0.0 | 0 |
|  | 'ER3-4' | 6.6 | 2.5 | 0.1 | 0.0 | 0 |
|  | 'HL1-2' | 4.0 | 2.3 | 0.2 | 1.2 | 0.1371 |
|  | 'HL2-3' | 3.3 | 2.6 | 0.2 | 1.2 | 0.12 |
|  | 'HL3-4' | 1.4 | 2.2 | 0.0 | 0.3 | 0 |
|  | 'HR1-2' | 18.4 | 3.1 | 0.4 | 0.0 | 0 |
|  | 'HR2-3' | 7.2 | 2.4 | 0.0 | 0.0 | 0 |
|  | 'HR3-4' | 1.6 | 2.3 | 0.0 | 0.1 | 0 |
|  | 'PL1-2' | 17.6 | 2.7 | 0.1 | 0.5 | 0.0286 |
|  | 'PL2-3' | 3.3 | 2.5 | 0.1 | 0.5 | 0.0571 |
|  | 'PL3-4' | 1.6 | 2.0 | 0.0 | 0.0 | 0 |
|  | 'PR1-2' | 117.1 | 14.0 | 9.4 | 5.01714286 | 3.5886 |
|  | 'PR2-3' | 25.3 | 3.4 | 0.7 | 5.05 | 0.3714 |
|  | 'PR3-4' | 5.8 | 2.0 | 0.1 | 0.4 | 0.0057 |
| Threshold |  |  |  |  | 5.03 | 1.3 |

| **Patient 6** | **Channels** | **Ripples** | **FR** | **FRandR** | **Spikes** | **S + HFO** |
| --- | --- | --- | --- | --- | --- | --- |
| **ILAE 1** | **'AL1-2'** | 83.8 | 9.2 | 4.3 | 0.1 | 0.057 |
|  | **'AL2-3'** | 55.9 | 5.5 | 2.4 | 0.1 | 0.051 |
|  | **'AL3-4'** | 5.3 | 2.6 | 0.0 | 0.1 | 0.006 |
|  | **'AR1-2'** | 27.0 | 8.6 | 4.0 | 3.1 | 1.154 |
|  | **'AR2-3'** | 20.3 | 6.2 | 3.2 | 3.1 | 1.594 |
|  | **'AR3-4'** | 2.8 | 2.7 | 0.1 | 0.5 | 0.040 |
|  | **'EL1-2'** | 2.3 | 2.3 | 0.0 | 0.0 | 0.000 |
|  | **'EL2-3'** | 1.5 | 2.6 | 0.0 | 0.0 | 0.000 |
|  | **'EL3-4'** | 0.6 | 2.3 | 0.0 | 0.1 | 0.000 |
|  | **'ER1-2'** | 13.6 | 3.6 | 0.9 | 0.1 | 0.029 |
|  | **'ER2-3'** | 9.6 | 3.0 | 0.5 | 0.1 | 0.023 |
|  | **'ER3-4'** | 0.7 | 2.2 | 0.0 | 0.2 | 0.000 |
|  | **'HL1-2'** | 0.1 | 2.3 | 0.0 | 0.0 | 0.000 |
|  | **'HL2-3'** | 0.1 | 2.2 | 0.0 | 0.0 | 0.000 |
|  | **'HL3-4'** | 0.0 | 2.6 | 0.0 | 0.2 | 0.000 |
|  | **'HR1-2'** | 0.4 | 2.4 | 0.0 | 0.0 | 0.006 |
|  | **'HR2-3'** | 0.3 | 2.5 | 0.0 | 0.0 | 0.006 |
|  | **'HR3-4'** | 0.2 | 2.7 | 0.0 | 0.2 | 0.006 |
|  | **'PL1-2'** | 45.4 | 2.4 | 0.1 | 0.0 | 0.000 |
|  | **'PL2-3'** | 76.3 | 2.2 | 0.4 | 0.0 | 0.000 |
|  | **'PL3-4'** | 1.9 | 2.4 | 0.0 | 0.0 | 0.000 |
|  | **'PR1-2'** | 147.3 | 18.1 | 7.6 | 4.72571429 | 2.000 |
|  | **'PR2-3'** | 14.2 | 11.6 | 2.8 | 4.7 | 1.457 |
|  | **'PR3-4'** | 0.5 | 2.7 | 0.2 | 0.6 | 0.017 |
| Threshold |  |  |  |  | 4.73 | 1.72 |

| **Patient 7** | **Channels** | **Ripples** | **FR** | **FRandR** | **Spikes** | **S + HFO** |
| --- | --- | --- | --- | --- | --- | --- |
| **ILAE 3** | **'AR1-2'** | 4.4 | 5.2 | 0.4 | 2.6 | 0.2 |
|  | **'AR2-3'** | 3.0 | 3.4 | 0.0 | 2.6 | 0 |
|  | **'AR3-4'** | 2.6 | 0.6 | 0.0 | 0.2 | 0 |
|  | **'AL1-2'** | 11.0 | 5.6 | 1.0 | 0.2 | 0 |
|  | **'AL2-3'** | 3.8 | 3.4 | 0.0 | 0.4 | 0 |
|  | **'AL3-4'** | 0.6 | 3.6 | 0.0 | 0.2 | 0 |
|  | **'HR1-2'** | 42.6 | 17.4 | 6.0 | 6.4 | 0.8 |
|  | **'HR2-3'** | 69.0 | 21.8 | 7.6 | 8.6 | 1.4 |
|  | **'HR3-4'** | 75.6 | 27.0 | 7.6 | 8.6 | 2.4 |
|  | **'HL1-2'** | 15.8 | 36.2 | 7.4 | 0.2 | 0 |
|  | **'HL2-3'** | 4.8 | 18.0 | 1.6 | 0.2 | 0 |
|  | **'HL3-4'** | 1.2 | 6.0 | 0.0 | 0.2 | 0 |
|  | **'ER1-2'** | 1.0 | 3.4 | 0.0 | 0.0 | 0 |
|  | **'ER2-3'** | 2.4 | 3.8 | 0.0 | 0.0 | 0 |
|  | **'ER3-4'** | 2.4 | 5.2 | 0.0 | 0.2 | 0 |
|  | **'EL1-2'** | 22.4 | 5.0 | 0.6 | 0.0 | 0 |
|  | **'EL2-3'** | 1.4 | 2.0 | 0.0 | 0.0 | 0 |
|  | **'EL3-4'** | 6.2 | 3.2 | 0.0 | 0.0 | 0 |
|  | **'PR1-2'** | 11.8 | 6.0 | 2.0 | 2.4 | 1.6 |
|  | **'PR2-3'** | 23.8 | 6.2 | 2.2 | 2.4 | 1.4 |
|  | **'PR3-4'** | 20.6 | 4.6 | 1.4 | 0.8 | 0.4 |
|  | **'PL1-2'** | 72.2 | 7.8 | 4.0 | 1.4 | 0 |
|  | **'PL2-3'** | 57.6 | 17.4 | 8.2 | 1.4 | 0.8 |
|  | **'PL3-4'** | 3.6 | 5.0 | 0.2 | 0.4 | 0 |
| Threshold |  |  |  |  | 8.60 | 1.8 |

| **Patient 8** | **Channels** | **Ripples** | **FR** | **FRandR** | **Spikes** | **S + HFO** |
| --- | --- | --- | --- | --- | --- | --- |
| **ILAE 3** | **'AL1-2'** | 18.4 | 11.5 | 7.4 | 0.4625 | 0.2 |
|  | **'AL2-3'** | 1.9 | 3.1 | 0.0 | 0.5 | 0.0125 |
|  | **'AL3-4'** | 0.1 | 1.9 | 0.0 | 0.3 | 0 |
|  | **'AR1-2'** | 4.6 | 2.9 | 0.1 | 0.0 | 0 |
|  | **'AR2-3'** | 6.2 | 2.2 | 0.1 | 0.0 | 0 |
|  | **'AR3-4'** | 1.7 | 2.4 | 0.0 | 0.1 | 0 |
|  | **'EL1-2'** | 3.4 | 5.9 | 0.8 | 0.1 | 0.025 |
|  | **'EL2-3'** | 1.1 | 2.2 | 0.0 | 0.1 | 0 |
|  | **'EL3-4'** | 0.3 | 2.4 | 0.0 | 0.2 | 0 |
|  | **'ER1-2'** | 2.6 | 4.8 | 1.1 | 0.0 | 0 |
|  | **'ER2-3'** | 2.8 | 3.5 | 0.6 | 0.0 | 0 |
|  | **'ER3-4'** | 2.6 | 2.2 | 0.1 | 0.2 | 0 |
|  | **'HL1-2'** | 4.1 | 2.3 | 0.0 | 0.0 | 0 |
|  | **'HL2-3'** | 1.7 | 2.4 | 0.0 | 0.0 | 0 |
|  | **'HL3-4'** | 0.5 | 2.1 | 0.0 | 0.2 | 0 |
|  | **'HR1-2'** | 3.9 | 4.7 | 1.5 | 0.1 | 0.075 |
|  | **'HR2-3'** | 4.4 | 5.2 | 1.7 | 0.1 | 0.1 |
|  | **'HR3-4'** | 0.8 | 2.8 | 0.2 | 0.2 | 0.0125 |
|  | **'PL1-2'** | 2.5 | 3.8 | 0.5 | 0.0 | 0 |
|  | **'PL2-3'** | 0.1 | 1.7 | 0.0 | 0.0 | 0 |
|  | **'PL3-4'** | 0.1 | 2.1 | 0.0 | 0.2 | 0 |
|  | **'PR1-2'** | 1.8 | 3.8 | 0.7 | 0.0 | 0 |
|  | **'PR2-3'** | 0.3 | 2.2 | 0.1 | 0.0 | 0 |
|  | **'PR3-4'** | 0.2 | 2.3 | 0.0 | 0.0 | 0 |
| Threshold |  |  |  |  | 0.46 | 0.13 |

| **Patient 9** | **Channels** | **Ripples** | **FR** | **FRandR** | **Spikes** | **S + HFO** |
| --- | --- | --- | --- | --- | --- | --- |
| **ILAE 5** | **'AL1-2'** | 35.7 | 11.5 | 7.0 | 3.2 | 1.3167 |
|  | **'AL2-3'** | 12.8 | 5.5 | 0.8 | 3.2 | 0.1333 |
|  | **'AL3-4'** | 3.6 | 5.3 | 1.0 | 0.4 | 0 |
|  | **'AR1-2'** | 22.9 | 5.5 | 2.6 | 2.8 | 1.5833 |
|  | **'AR2-3'** | 7.3 | 2.2 | 0.1 | 2.8 | 0.1 |
|  | **'AR3-4'** | 1.6 | 2.5 | 0.0 | 1.3 | 0 |
|  | **'EL1-2'** | 59.0 | 4.5 | 1.5 | 4.2 | 0.4 |
|  | **'EL2-3'** | 27.2 | 2.7 | 0.2 | 4.2 | 0.1 |
|  | **'EL3-4'** | 1.9 | 2.0 | 0.0 | 0.3 | 0 |
|  | **'ER1-2'** | 22.0 | 5.8 | 1.1 | 3.6 | 0.2833 |
|  | **'ER2-3'** | 9.9 | 1.9 | 0.1 | 3.6 | 0.0667 |
|  | **'ER3-4'** | 6.8 | 2.3 | 0.0 | 0.5 | 0 |
|  | **'HL1-2'** | 64.7 | 42.5 | 27.5 | 1.93333333 | 0.9667 |
|  | **'HL2-3'** | 14.4 | 2.2 | 0.2 | 2.0 | 0.1167 |
|  | **'HL3-4'** | 2.9 | 1.9 | 0.0 | 0.2 | 0 |
|  | **'HR1-2'** | 23.3 | 8.5 | 4.0 | 4.4 | 1.1167 |
|  | **'HR2-3'** | 23.3 | 2.6 | 0.8 | 4.6 | 0.6833 |
|  | **'HR3-4'** | 15.8 | 2.3 | 0.1 | 1.9 | 0.0333 |
|  | **'PL1-2'** | 28.3 | 11.1 | 5.9 | 4.3 | 1.6 |
|  | **'PL2-3'** | 14.5 | 5.5 | 1.2 | 4.3 | 0.7167 |
|  | **'PL3-4'** | 6.6 | 3.2 | 0.2 | 0.0 | 0 |
|  | **'PR1-2'** | 52.5 | 6.2 | 3.6 | 3.8 | 1.65 |
|  | **'PR2-3'** | 6.7 | 2.0 | 0.2 | 3.8 | 0.2333 |
|  | **'PR3-4'** | 4.9 | 2.0 | 0.1 | 0.3 | 0 |
| Threshold |  |  |  |  | 4.44 | 1.615 |

| **Patient 10** | **Channels** | **Ripples** | **FR** | **FRandR** | **Spikes** | **S + HFO** |
| --- | --- | --- | --- | --- | --- | --- |
| **ILAE 1** | **'GR1-2'** | 9 | 3 | 0 | 0.0 | 0 |
|  | **'GR2-3'** | 14 | 1 | 0 | 0.0 | 0 |
|  | **'GR3-4'** | 11 | 2 | 0 | 0.0 | 0 |
|  | **'GR4-5'** | 4 | 2 | 0 | 0.0 | 0 |
|  | **'GR5-6'** | 2 | 2 | 0 | 0.0 | 0 |
|  | **'GR6-7'** | 29 | 3 | 0 | 0.0 | 0 |
|  | **'GR7-8'** | 57 | 3 | 0 | 0.0 | 0 |
|  | **'GR9-10'** | 42 | 3 | 0 | 0.0 | 0 |
|  | **'GR10-11'** | 44 | 2 | 0 | 0.0 | 0 |
|  | **'GR11-12'** | 60 | 2 | 0 | 0.1 | 0 |
|  | **'GR12-13'** | 38 | 3 | 0 | 0.1 | 0 |
|  | **'GR13-14'** | 25 | 3 | 0 | 0.1 | 0 |
|  | **'GR14-15'** | 35 | 3 | 0 | 0.4 | 0 |
|  | **'GR15-16'** | 61 | 4 | 1 | 0.5 | 0.0333 |
|  | **'GR17-18'** | 5 | 3 | 0 | 0.0 | 0 |
|  | **'GR18-19'** | 13 | 3 | 0 | 0.0 | 0 |
|  | **'GR19-20'** | 9 | 2 | 0 | 0.0 | 0 |
|  | **'GR20-21'** | 3 | 2 | 0 | 0.0 | 0 |
|  | **'GR21-22'** | 8 | 2 | 0 | 0.0 | 0 |
|  | **'GR22-23'** | 14 | 3 | 0 | 0.1 | 0 |
|  | **'GR23-24'** | 96 | 3 | 1 | 0.1 | 0 |
|  | **'GR25-26'** | 0 | 3 | 0 | 0.0 | 0 |
|  | **'GR26-27'** | 2 | 3 | 0 | 0.0 | 0 |
|  | **'GR27-28'** | 3 | 2 | 0 | 0.0 | 0 |
|  | **'GR28-29'** | 27 | 2 | 0 | 0.0 | 0.0 |
|  | **'GR29-30'** | 37 | 2 | 0 | 0.2 | 0.0 |
|  | **'GR30-31'** | 31 | 2 | 0 | 0.2 | 0.0 |
|  | **'GR31-32'** | 22 | 2 | 0 | 0.0 | 0.0 |
|  | **'IAR1-2'** | 5 | 2 | 0 | 0.0 | 0.0 |
|  | **'IAR2-3'** | 5 | 2 | 0 | 0.0 | 0.0 |
|  | **'IAR3-4'** | 0 | 2 | 0 | 0.0 | 0.0 |
|  | **'IPR1-2'** | 131 | 12 | 5 | 1.76666667 | 0.100 |
|  | **'IPR2-3'** | 94 | 11 | 4 | 1.76666667 | 0.1333 |
|  | **'IPR3-4'** | 16 | 3 | 0 | 0 | 0 |
| Threshold |  |  |  |  | 1.513 | 0.0867 |

| **Patient 11** | **Channels** | **Ripples** | **FR** | **FRandR** | **Spikes2** | **S2+HFO** |
| --- | --- | --- | --- | --- | --- | --- |
| **ILAE 1** | **'GR1-2'** | 75.1 | 4.6 | 1.4 | 0 | 0 |
|  | **'GR2-3'** | 44.8 | 2.6 | 0.4 | 0 | 0 |
|  | **'GR3-4'** | 65.5 | 2.6 | 0.4 | 0 | 0 |
|  | **'GR4-5'** | 107.3 | 2.8 | 0.6 | 0.0316 | 0 |
|  | **'GR5-6'** | 142.1 | 3.4 | 1.0 | 0.1053 | 0 |
|  | **'GR6-7'** | 84.7 | 3.6 | 0.7 | 0.0737 | 0 |
|  | **'GR7-8'** | 24.0 | 2.2 | 0.1 | 0 | 0 |
|  | **'GR9-10'** | 139.3 | 6.7 | 3.0 | 0.0947 | 0.0105 |
|  | **'GR10-11'** | 144.9 | 6.3 | 2.5 | 0.1789 | 0 |
|  | **'GR11-12'** | 146.6 | 3.9 | 1.2 | 0.2 | 0.0105 |
|  | **'GR12-13'** | 167.8 | 4.2 | 1.4 | 0.1368 | 0 |
|  | **'GR13-14'** | 145.6 | 3.6 | 1.1 | 0.0842 | 0 |
|  | **'GR14-15'** | 103.4 | 3.4 | 0.7 | 0.0737 | 0.0105 |
|  | **'GR15-16'** | 85.2 | 3.3 | 0.5 | 0.0316 | 0 |
|  | **'GR17-18'** | 106.9 | 5.9 | 2.3 | 1.0421 | 0.1579 |
|  | **'GR18-19'** | 96.2 | 4.6 | 1.6 | 1.0632 | 0.0421 |
|  | **'GR19-20'** | 82.6 | 3.0 | 0.4 | 0.1158 | 0 |
|  | **'GR20-21'** | 136.9 | 3.7 | 1.2 | 0.1053 | 0 |
|  | **'GR21-22'** | 156.7 | 3.7 | 1.4 | 0.0316 | 0 |
|  | **'GR22-23'** | 58.0 | 2.5 | 0.3 | 0 | 0 |
|  | **'GR23-24'** | 84.3 | 3.0 | 0.6 | 0 | 0 |
|  | **'GR25-26'** | 56.2 | 15.5 | 4.0 | 4.9789 | 0.6105 |
|  | **'GR26-27'** | 84.8 | 22.4 | 4.7 | 5.4 | 0.5579 |
|  | **'GR27-28'** | 70.7 | 10.4 | 2.8 | 2.7579 | 0.2842 |
|  | **'GR28-29'** | 42.2 | 4.5 | 1.2 | 0.0211 | 0 |
|  | **'GR29-30'** | 54.8 | 3.6 | 0.6 | 0.0211 | 0 |
|  | **'GR30-31'** | 37.4 | 2.4 | 0.2 | 0 | 0 |
|  | **'GR31-32'** | 28.8 | 2.4 | 0.1 | 0 | 0 |
|  | **'GR33-34'** | 61.3 | 11.3 | 2.6 | 9.3263 | 0.5368 |
|  | **'GR34-35'** | 68.4 | 36.4 | 4.1 | 11.5579 | 0.5158 |
|  | **'GR35-36'** | 66.8 | 34.7 | 5.5 | 9.4 | 0.9474 |
|  | **'GR36-37'** | 57.5 | 6.6 | 1.1 | 0.0211 | 0 |
|  | **'GR37-38'** | 77.9 | 6.6 | 1.5 | 0 | 0 |
|  | **'GR38-39'** | 48.5 | 2.7 | 0.4 | 0.0526 | 0 |
|  | **'GR39-40'** | 40.7 | 2.4 | 0.1 | 0.0316 | 0 |
|  | **'GR41-42'** | 52.4 | 6.6 | 1.7 | 2.7789 | 0.1579 |
|  | **'GR42-43'** | 45.0 | 9.0 | 2.3 | 2.7789 | 0.1263 |
|  | **'GR43-44'** | 25.9 | 2.6 | 0.4 | 1.9368 | 0.1263 |
|  | **'GR44-45'** | 63.5 | 4.8 | 1.2 | 0 | 0 |
|  | **'GR45-46'** | 114.5 | 5.1 | 1.1 | 0 | 0 |
|  | **'GR46-47'** | 104.9 | 3.6 | 0.8 | 0 | 0 |
|  | **'GR47-48'** | 56.3 | 2.7 | 0.3 | 0 | 0 |
|  | **'GR49-50'** | 68.5 | 5.3 | 1.5 | 0 | 0 |
|  | **'GR50-51'** | 51.7 | 4.2 | 0.9 | 0 | 0 |
|  | **'GR51-52'** | 54.7 | 4.2 | 0.9 | 0 | 0 |
|  | **'GR52-53'** | 5.3 | 2.6 | 0.0 | 0 | 0 |
|  | **'GR53-54'** | 14.4 | 2.4 | 0.0 | 0 | 0 |
|  | **'GR54-55'** | 32.1 | 2.3 | 0.1 | 0 | 0.0 |
|  | **'GR55-56'** | 38.1 | 2.7 | 0.1 | 0 | 0 |
|  | **'GR57-58'** | 78.5 | 3.3 | 0.5 | 0 | 0 |
|  | **'GR58-59'** | 70.5 | 2.6 | 0.3 | 0 | 0 |
|  | **'GR59-60'** | 38.1 | 2.2 | 0.1 | 0 | 0 |
|  | **'GR60-61'** | 16.6 | 2.0 | 0.0 | 0 | 0 |
|  | **'GR61-62'** | 51.6 | 3.1 | 0.4 | 0 | 0 |
|  | **'GR62-63'** | 87.1 | 3.1 | 0.6 | 0.0105 | 0 |
|  | **'GR63-64'** | 60.2 | 2.4 | 0.3 | 0.0105 | 0 |
|  | **'TR1-2'** | 121.5 | 77.6 | 31.4 | 1.6737 | 0.8632 |
|  | **'TR2-3'** | 109.0 | 60.7 | 22.5 | 2.5684 | 0.9684 |
|  | **'TR3-4'** | 55.1 | 17.6 | 6.5 | 2.0947 | 0.4421 |
|  | **'TR4-5'** | 42.2 | 4.2 | 0.9 | 1.3789 | 0.0842 |
|  | **'TR5-6'** | 23.5 | 3.3 | 0.4 | 0.4316 | 0.0105 |
|  | **'TR6-7'** | 20.6 | 2.7 | 0.2 | 0.4316 | 0 |
|  | **'TR7-8'** | 0.2 | 2.6 | 0.0 | 0.4316 | 0 |
|  | **'TR8-9'** | 0.1 | 2.3 | 0.0 | 0 | 0 |
|  | **'TR9-10'** | 0.2 | 2.7 | 0.0 | 0 | 0 |
| Threshold |  |  |  |  | 6.3816 | 0.6737 |

| **Patient 12** | **Channels** | **Ripples** | **FR** | **FRandR** | **Spikes2** | **S2 + HFO** |
| --- | --- | --- | --- | --- | --- | --- |
| **ILAE 1** | **'GL1-2'** | 60.9 | 6.7 | 0.7 | 0.144 | 0 |
|  | **'GL2-3'** | 42.8 | 2.9 | 0.2 | 0.256 | 0 |
|  | **'GL3-4'** | 53.1 | 2.8 | 0.3 | 0.208 | 0 |
|  | **'GL4-5'** | 41.1 | 2.7 | 0.3 | 0.088 | 0 |
|  | **'GL5-6'** | 15.9 | 2.4 | 0.0 | 0.008 | 0 |
|  | **'GL6-7'** | 53.4 | 2.7 | 0.7 | 0.008 | 0 |
|  | **'GL7-8'** | 72.6 | 3.5 | 1.0 | 0.008 | 0 |
|  | **'GL9-10'** | 58.9 | 3.2 | 0.3 | 0.936 | 0.008 |
|  | **'GL10-11'** | 61.8 | 3.3 | 0.5 | 1.488 | 0.04 |
|  | **'GL11-12'** | 45.0 | 2.8 | 0.3 | 0.936 | 0.024 |
|  | **'GL12-13'** | 10.4 | 2.2 | 0.0 | 0.184 | 0 |
|  | **'GL13-14'** | 38.2 | 2.9 | 0.4 | 0.112 | 0 |
|  | **'GL14-15'** | 50.3 | 3.0 | 0.5 | 0 | 0 |
|  | **'GL15-16'** | 35.1 | 2.3 | 0.1 | 0.016 | 0 |
|  | **'GL17-18'** | 61.4 | 3.1 | 0.4 | 0.44 | 0.008 |
|  | **'GL18-19'** | 43.5 | 2.7 | 0.2 | 1.048 | 0 |
|  | **'GL19-20'** | 45.0 | 2.5 | 0.2 | 1.032 | 0.016 |
|  | **'GL20-21'** | 52.5 | 3.2 | 0.4 | 0.96 | 0 |
|  | **'GL21-22'** | 12.6 | 2.7 | 0.0 | 0.712 | 0 |
|  | **'GL22-23'** | 29.1 | 2.6 | 0.3 | 0.008 | 0 |
|  | **'GL23-24'** | 37.4 | 2.4 | 0.3 | 0.024 | 0 |
|  | **'GL25-26'** | 42.8 | 4.0 | 0.4 | 1.368 | 0.024 |
|  | **'GL26-27'** | 35.8 | 3.1 | 0.3 | 1.368 | 0.04 |
|  | **'GL27-28'** | 36.6 | 3.1 | 0.2 | 0 | 0 |
|  | **'GL28-29'** | 34.1 | 2.9 | 0.2 | 0.112 | 0 |
|  | **'GL29-30'** | 40.5 | 3.3 | 0.2 | 0.448 | 0.008 |
|  | **'GL30-31'** | 59.1 | 2.9 | 0.4 | 0.48 | 0 |
|  | **'GL31-32'** | 122.2 | 3.7 | 1.5 | 0.096 | 0 |
|  | **'TL1-2'** | 19.8 | 4.1 | 1.7 | 0.344 | 0.104 |
|  | **'TL2-3'** | 74.9 | 3.7 | 1.4 | 0.488 | 0.144 |
|  | **'TL3-4'** | 67.6 | 2.8 | 0.7 | 0.528 | 0.056 |
|  | **'TL4-5'** | 36.4 | 4.2 | 0.9 | 0.432 | 0.08 |
|  | **'TL5-6'** | 28.7 | 4.2 | 0.9 | 0.304 | 0.064 |
|  | **'TL6-7'** | 8.8 | 2.9 | 0.1 | 0.056 | 0 |
|  | **'TL7-8'** | 2.3 | 2.1 | 0.0 | 0.032 | 0 |
|  | **'TL8-9'** | 0.1 | 2.9 | 0.0 | 0 | 0 |
|  | **'TL9-10'** | 0.1 | 2.9 | 0.0 | 0 | 0 |
| Threshold |  |  |  |  | 1.368 | 0.0956 |

| **Patient 13** | **Channels** | **Ripples** | **FR** | **FRandR** | **Spikes** | **S + HFO** |
| --- | --- | --- | --- | --- | --- | --- |
| **ILAE 1** | **'GR1-2'** | 0.00 | 2.7 | 0.6 | 0.00 | 0 |
|  | **'GR2-3'** | 0.00 | 2.5 | 0.5 | 0.00 | 0 |
|  | **'GR3-4'** | 0.00 | 2.9 | 0.7 | 0.00 | 0 |
|  | 'GR4-5' | 0.00 |  |  | 0.00 | 0 |
|  | 'GR5-6' | 0.00 |  |  | 0.00 | 0 |
|  | 'GR6-7' | 0.00 |  |  | 0.00 | 0 |
|  | 'GR7-8' | 0.18 |  |  | 0.18 | 0 |
|  | **'GR9-10'** | 0.00 | 2.0 | 0.0 | 0.00 | 0 |
|  | **'GR10-11'** | 0.00 | 2.2 | 0.1 | 0.00 | 0 |
|  | **'GR11-12'** | 0.00 | 2.4 | 0.6 | 0.00 | 0 |
|  | 'GR12-13' | 0.00 |  |  | 0.00 | 0 |
|  | 'GR13-14' | 0.01 |  |  | 0.01 | 0 |
|  | 'GR14-15' | 0.03 |  |  | 0.03 | 0 |
|  | 'GR15-16' | 0.19 |  |  | 0.19 | 0 |
|  | **'GR17-18'** | 0.00 | 3.8 | 1.6 | 0.00 | 0 |
|  | **'GR18-19'** | 0.00 | 4.3 | 1.5 | 0.00 | 0 |
|  | **'GR19-20'** | 0.00 | 2.7 | 0.5 | 0.00 | 0 |
|  | 'GR20-21' | 0.04 |  |  | 0.04 | 0 |
|  | 'GR21-22' | 0.04 |  |  | 0.04 | 0 |
|  | 'GR22-23' | 0.00 |  |  | 0.00 | 0 |
|  | 'GR23-24' | 0.10 |  |  | 0.10 | 0 |
|  | **'GR25-26'** | 0.00 | 3.7 | 1.1 | 0.00 | 0 |
|  | **'GR26-27'** | 0.05 | 3.3 | 1.0 | 0.05 | 0 |
|  | **'GR27-28'** | 0.14 | 3.7 | 1.4 | 0.14 | 0 |
|  | **'GR28-29'** | 194.7 | 3.2 | 1.4 | 0.10 | 0 |
|  | **'GR29-30'** | 210.5 | 6.0 | 3.0 | 0.01 | 0 |
|  | **'GR30-31'** | 181.8 | 3.7 | 1.4 | 0.01 | 0 |
|  | **'GR31-32'** | 148.3 | 5.6 | 1.7 | 0.09 | 0 |
|  | **'GR33-34'** | 121.4 | 3.4 | 0.8 | 0.00 | 0 |
|  | **'GR34-35'** | 193.4 | 4.0 | 1.6 | 0.24 | 0.0125 |
|  | **'GR35-36'** | 163.7 | 4.8 | 1.9 | 0.25 | 0 |
|  | **'GR36-37'** | 141.2 | 3.5 | 1.0 | 0.05 | 0 |
|  | **'GR37-38'** | 60.2 | 2.4 | 0.3 | 0.00 | 0 |
|  | **'GR38-39'** | 73.3 | 2.6 | 0.4 | 0.00 | 0 |
|  | **'GR39-40'** | 80.1 | 3.0 | 0.4 | 0.09 | 0 |
|  | **'GR41-42'** | 116.2 | 4.6 | 1.2 | 0.01 | 0 |
|  | **'GR42-43'** | 157.4 | 5.3 | 1.9 | 0.09 | 0 |
|  | **'GR43-44'** | 171.0 | 4.5 | 1.7 | 0.15 | 0.0125 |
|  | **'GR44-45'** | 197.2 | 5.0 | 2.1 | 0.13 | 0 |
|  | **'GR45-46'** | 200.8 | 4.1 | 1.6 | 0.06 | 0.0125 |
|  | **'GR46-47'** | 183.8 | 4.4 | 1.8 | 0.01 | 0 |
|  | **'GR47-48'** | 130.9 | 3.8 | 1.1 | 0.20 | 0 |
|  | **'GR49-50'** | 91.1 | 3.6 | 0.7 | 0.00 | 0 |
|  | **'GR50-51'** | 109.7 | 3.3 | 0.7 | 0.00 | 0 |
|  | **'GR51-52'** | 157.3 | 4.7 | 1.4 | 0.00 | 0 |
|  | **'GR52-53'** | 190.2 | 4.9 | 2.1 | 0.00 | 0 |
|  | **'GR53-54'** | 189.6 | 4.0 | 1.4 | 0.00 | 0 |
|  | **'GR54-55'** | 173.2 | 2.7 | 0.8 | 0.00 | 0 |
|  | **'GR55-56'** | 106.1 | 2.6 | 0.5 | 0.200 | 0 |
|  | **'GR57-58'** | 90.4 | 3.2 | 0.7 | 0.000 | 0 |
|  | **'GR58-59'** | 110.5 | 4.3 | 0.8 | 0.000 | 0 |
|  | **'GR59-60'** | 130.5 | 3.7 | 1.3 | 0.000 | 0 |
|  | **'GR60-61'** | 195.9 | 5.2 | 2.3 | 0.000 | 0 |
|  | **'GR61-62'** | 169.9 | 5.2 | 2.2 | 0.000 | 0 |
|  | **'GR62-63'** | 144.9 | 3.5 | 1.2 | 0.000 | 0 |
|  | **'GR63-64'** | 121.0 | 3.7 | 1.3 | 0.000 | 0 |
|  | **'TR1-2'** | 23.3 | 9.7 | 3.9 | 4.138 | 0.6125 |
|  | **'TR2-3'** | 30.9 | 12.4 | 4.2 | 16.438 | 2.1375 |
|  | **'TR3-4'** | 43.7 | 33.9 | 5.0 | 16.463 | 0.95 |
|  | **'TR4-5'** | 29.2 | 10.7 | 3.5 | 0.050 | 0 |
|  | **'TR5-6'** | 53.8 | 2.7 | 0.3 | 0.225 | 0 |
|  | **'TR6-7'** | 65.0 | 2.6 | 0.5 | 0.000 | 0 |
|  | **'TR7-8'** | 47.8 | 2.2 | 0.2 | 0.000 | 0 |
|  | **'TR8-9'** | 7.4 | 1.9 | 0.0 | 0.000 | 0 |
|  | **'TR9-10'** | 14.0 | 3.5 | 0.6 | 0.000 | 0 |
| Threshold |  |  |  |  | 1.2219 | 0.1625 |

| **Patient 14** | **Channels** | **Ripples** | **FR** | **FRandR** | **Spikes** | **S + HFO** |
| --- | --- | --- | --- | --- | --- | --- |
| **ILAE 1** | **'IAR1-2'** | 76.0 | 4.3 | 1.4 | 0.00 | 0 |
|  | **'IAR2-3'** | 57.5 | 4.4 | 1.2 | 0.00 | 0 |
|  | **IAR3-4'** |  |  |  | 0.63076923 | 0.0769 |
|  | **IAR4-5'** |  |  |  | 1.01538462 | 0.2 |
|  | **IAR5-6'** |  |  |  | 0.69230769 | 0.1077 |
|  | **'IPR1-2'** | 88.8 | 2.6 | 0.5 | 0.47692308 | 0 |
|  | **'IPR2-3'** | 78.8 | 4.9 | 1.0 | 2.16923077 | 0.0308 |
|  | **'IPR3-4'** | 142.7 | 47.0 | 17.2 | 2.29230769 | 0.3692 |
|  | **'PLR1-2'** | 17.5 | 3.0 | 0.2 | 0.00 | 0 |
|  | **'PLR2-3'** | 14.8 | 2.4 | 0.2 | 0.00 | 0 |
|  | **'PLR3-4'** | 56.8 | 3.0 | 0.4 | 0.00 | 0 |
|  | **'PLR4-5'** | 80.4 | 3.1 | 0.7 | 0.00 | 0 |
|  | **PLR5-6'** |  |  |  | 0.00 | 0 |
|  | **PLR6-7'** |  |  |  | 0.00 | 0 |
|  | **PLR7-8'** |  |  |  | 0.20 | 0.0308 |
|  | **'PLR9-10'** | 38.6 | 2.7 | 0.2 | 0.00 | 0 |
|  | **'PLR10-11'** | 71.2 | 2.6 | 0.5 | 0.00 | 0 |
|  | **'PLR11-12'** | 64.1 | 3.1 | 0.5 | 0.00 | 0 |
|  | **'PLR12-13'** | 26.0 | 2.5 | 0.3 | 0.00 | 0 |
|  | **PLR13-14'** |  |  |  | 0.00 | 0 |
|  | **PLR14-15'** |  |  |  | 0.00 | 0 |
|  | **PLR15-16'** |  |  |  | 0.08 | 0 |
|  | **'PMR1-2'** | 103.1 | 5.0 | 2.0 | 0.00 | 0 |
|  | **'PMR2-3'** | 98.5 | 5.9 | 2.8 | 0.00 | 0 |
|  | **'PMR3-4'** | 106.4 | 5.6 | 2.1 | 0.14 | 0.0154 |
|  | **'PMR4-5'** | 84.4 | 7.5 | 1.9 | 0.15 | 0.0308 |
|  | **PMR5-6'** |  |  |  | 0.03 | 0.0154 |
|  | **PMR6-7'** |  |  |  | 0.05 | 0 |
|  | **PMR7-8'** |  |  |  | 0.20 | 0 |
|  | **'PMR9-10'** | 92.9 | 5.3 | 2.4 | 0.00 | 0 |
|  | **'PMR10-11'** | 77.5 | 2.6 | 0.8 | 0.02 | 0 |
|  | **'PMR11-12'** | 86.2 | 4.0 | 1.1 | 0.03 | 0 |
|  | **'PMR12-13'** | 85.2 | 6.2 | 2.4 | 0.02 | 0 |
|  | **PMR13-14'** |  |  |  | 0.00 | 0 |
|  | **PMR14-15'** |  |  |  | 0.00 | 0 |
|  | **PMR15-16'** |  |  |  | 0.20 | 0 |
|  | **'TR1-2'** | 8.1 | 3.1 | 0.1 | 0.00 | 0 |
|  | **'TR2-3'** | 7.6 | 2.9 | 0.1 | 0.00 | 0 |
|  | **'TR3-4'** | 8.2 | 3.2 | 0.1 | 0.00 | 0 |
|  | **'TR4-5'** | 8.9 | 2.6 | 0.0 | 0.00 | 0 |
|  | **'TR5-6'** | 9.0 | 2.5 | 0.0 | 0.20 | 0 |
|  | **'TR6-7'** | 1.0 | 2.2 | 0.0 | 0.00 | 0 |
|  | **'TR7-8'** | 0.4 | 2.4 | 0.0 | 0.00 | 0 |
|  | **'TR8-9'** | 0.1 | 2.3 | 0.0 | 0.00 | 0 |
|  | **'TR9-10'** | 0.1 | 2.6 | 0.0 | 0.00 | 0 |
| Threshold |  |  |  |  | 1.3038 | 0.1308 |

| **Patient 15** | **Channels** | **Ripples** | **FR** | **FRandR** | **Spikes2** | **S2 + HFO** |
| --- | --- | --- | --- | --- | --- | --- |
| **ILAE 1** | **'TBAL1-2'** | 99.2 | 2.6 | 0.6 | 0.2286 | 0 |
|  | **'TBAL2-3'** | 53.0 | 3.2 | 0.3 | 8.6857 | 0.1071 |
|  | **'TBAL3-4'** | 22.7 | 7.7 | 1.3 | 18.2286 | 0.4857 |
|  | **'TBPL1-2'** | 166.8 | 9.7 | 1.5 | 0.0714 | 0 |
|  | **'TBPL2-3'** | 177.6 | 5.9 | 1.5 | 1.2286 | 0.0357 |
|  | **'TBPL3-4'** | 75.6 | 2.8 | 0.5 | 1.2 | 0.0214 |
|  | **'TLL1-2'** | 191.8 | 5.9 | 2.5 | 10.4214 | 0.5571 |
|  | **'TLL2-3'** | 158.1 | 19.0 | 1.5 | 14.9357 | 0.3071 |
|  | **'TLL3-4'** | 123.2 | 19.9 | 1.6 | 15.3071 | 0.3857 |
|  | **'TLL4-5'** | 137.3 | 3.7 | 0.9 | 13.7286 | 0.1143 |
|  | **'TLL5-6'** | 151.7 | 3.3 | 1.3 | 0.0286 | 0 |
|  | 'TLL6-7' |  |  |  | 0 | 0 |
|  | 'TLL7-8' |  |  |  | 0 | 0 |
|  | **'TLL9-10'** | 66.2 | 3.3 | 0.5 | 1.3643 | 0.0643 |
|  | **'TLL10-11'** | 80.8 | 3.5 | 0.7 | 12.65 | 0.2643 |
|  | **'TLL11-12'** | 145.4 | 3.5 | 1.1 | 16.0071 | 0.4 |
|  | **'TLL12-13'** | 115.4 | 3.0 | 0.9 | 15.7071 | 0.2786 |
|  | **'TLL13-14'** | 142.4 | 3.0 | 1.1 | 0.0286 | 0 |
|  | 'TLL14-15' |  |  |  | 0 | 0 |
|  | 'TLL15-16' |  |  |  | 0 | 0 |
|  | **'TLL17-18'** | 126.5 | 3.5 | 1.1 | 0.0357 | 0 |
|  | **'TLL18-19'** | 197.6 | 3.5 | 1.5 | 0.05 | 0.0143 |
|  | **'TLL19-20'** | 224.8 | 3.3 | 1.5 | 0.0357 | 0 |
|  | **'TLL20-21'** | 210.7 | 3.9 | 1.4 | 0.0071 | 0 |
|  | **'TLL21-22'** | 206.1 | 2.7 | 1.2 | 0 | 0 |
|  | 'TLL22-23' |  |  |  | 0.0143 | 0 |
|  | 'TLL23-24' |  |  |  | 0.0143 | 0 |
|  | **'TLL25-26'** | 75.5 | 4.1 | 0.9 | 0.1071 | 0.0071 |
|  | **'TLL26-27'** | 125.4 | 3.4 | 0.8 | 0.4143 | 0.0071 |
|  | **'TLL27-28'** | 130.2 | 5.1 | 1.4 | 26.4857 | 0.6357 |
|  | **'TLL28-29'** | 153.3 | 3.5 | 1.2 | 25.3 | 0.4786 |
|  | **'TLL29-30'** | 200.9 | 3.8 | 1.5 | 1.0786 | 0.0071 |
|  | **'TLL30-31'** | 209.6 | 3.6 | 1.5 | 0.0286 | 0 |
|  | 'TLL31-32' |  |  |  | 0 | 0 |
| Threshold |  |  |  |  | 23.8857 | 0.5429 |

| **Patient 16** | **Channels** | **Ripples** | **FR** | **FRandR** | **Spikes2** | **S2 + HFO** |
| --- | --- | --- | --- | --- | --- | --- |
| **ILAE 1** | **'GL1-2'** | 26.7 | 2.1 | 0.1 | 0 | 0 |
|  | **'GL2-3'** | 48.1 | 2.7 | 0.4 | 0.0118 | 0 |
|  | **'GL3-4'** | 48.0 | 2.8 | 0.5 | 0.0941 | 0 |
|  | **'GL4-5'** | 43.4 | 2.3 | 0.3 | 0.6353 | 0.0235 |
|  | **'GL5-6'** | 104.5 | 13.4 | 5.8 | 8.0706 | 1.1529 |
|  | **'GL6-7'** | 105.1 | 15.7 | 6.8 | 9.4471 | 1.5176 |
|  | **'GL7-8'** | 129.3 | 8.5 | 4.6 | 7.3412 | 0.8 |
|  | **'GL9-10'** | 58.4 | 3.4 | 0.8 | 0 | 0 |
|  | **'GL10-11'** | 103.0 | 3.8 | 1.3 | 0 | 0 |
|  | **'GL11-12'** | 96.9 | 4.3 | 1.5 | 0.2 | 0.0353 |
|  | **'GL12-13'** | 69.0 | 2.6 | 0.5 | 4.6471 | 0.1176 |
|  | **'GL13-14'** | 80.2 | 24.4 | 11.5 | 20.7176 | 5.7765 |
|  | **'GL14-15'** | 88.4 | 26.6 | 6.6 | 21.8471 | 2.6824 |
|  | **'GL15-16'** | 104.8 | 10.8 | 3.4 | 19.5765 | 1.6941 |
|  | **'GL17-18'** | 127.4 | 7.3 | 2.5 | 0.0353 | 0.0118 |
|  | **'GL18-19'** | 77.6 | 3.4 | 1.0 | 0.3765 | 0.0235 |
|  | **'GL19-20'** | 31.7 | 2.3 | 0.2 | 0.1412 | 0 |
|  | **'GL20-21'** | 94.7 | 4.6 | 1.3 | 2.6824 | 0.1529 |
|  | **'GL21-22'** | 108.3 | 9.2 | 2.1 | 11.2235 | 0.3529 |
|  | **'GL22-23'** | 86.7 | 26.0 | 4.1 | 19.0824 | 0.9412 |
|  | **'GL23-24'** | 96.1 | 24.5 | 4.0 | 17.7529 | 0.8824 |
|  | **'GL25-26'** | 80.7 | 4.9 | 2.0 | 0 | 0 |
|  | **'GL26-27'** | 26.0 | 2.8 | 0.2 | 0.0824 | 0 |
|  | **'GL27-28'** | 72.0 | 3.3 | 0.9 | 1.4 | 0.0471 |
|  | **'GL28-29'** | 110.0 | 8.9 | 3.1 | 5.7765 | 0.4471 |
|  | **'GL29-30'** | 116.0 | 10.3 | 4.4 | 10.8118 | 1.0706 |
|  | **'GL30-31'** | 85.9 | 5.0 | 1.9 | 0.1294 | 0.0353 |
|  | **'GL31-32'** | 91.7 | 4.4 | 1.9 | 0.0588 | 0.0235 |
|  | **'TL1-2'** | 57.7 | 64.6 | 35.6 | 24.2 | 9.8 |
|  | **'TL2-3'** | 49.7 | 41.4 | 15.3 | 25.2706 | 6.0706 |
|  | **'TL3-4'** | 63.7 | 12.6 | 7.7 | 19.0824 | 4.1765 |
|  | **'TL4-5'** | 54.3 | 5.0 | 2.7 | 13.7294 | 1.5294 |
|  | **'TL5-6'** | 48.4 | 2.6 | 0.7 | 4.6824 | 0.1176 |
|  | **'TL6-7'** | 0.3 | 2.9 | 0.0 | 0.1059 | 0 |
|  | **'TL7-8'** | 0.1 | 2.9 | 0.0 | 0.1059 | 0 |
|  | **'TL8-9'** | 0.1 | 2.6 | 0.0 | 0 | 0 |
|  | **'TL9-10'** | 0.1 | 2.6 | 0.0 | 0 | 0 |
| Threshold |  |  |  |  | 23.3765 | 5.9676 |

| **Patient 17** | **Channels** | **Ripples** | **FR** | **FRandR** | **Spikes** | **S + HFO** |
| --- | --- | --- | --- | --- | --- | --- |
| **ILAE 5** | **'FAR1-2'** | 15.8 | 0.0 | 0.0 | 0.00 | 0 |
|  | **'FAR2-3'** | 55.0 | 2.0 | 0.6 | 0.20 | 0 |
|  | **'FAR3-4'** | 94.4 | 6.2 | 2.0 | 0.40 | 0 |
|  | **'FAR4-5'** | 96.6 | 11.0 | 0.6 | 1.40 | 0.02352941 |
|  | **'FAR5-6'** | 77.4 | 21.8 | 1.0 | 6.20 | 1.15294118 |
|  | **'FAR6-7'** | 96.0 | 25.0 | 1.0 | 6.20 | 1.51764706 |
|  | **'FAR7-8'** | 105.0 | 16.0 | 0.4 | 3.60 | 0.8 |
|  | **'FAR9-10'** | 15.0 | 0.0 | 0.0 | 0.00 | 0 |
|  | **'FAR10-11'** | 33.6 | 2.8 | 0.6 | 0.00 | 0 |
|  | **'FAR11-12'** | 56.2 | 2.4 | 0.0 | 0.00 | 0.03529412 |
|  | **'FAR12-13'** | 82.6 | 17.4 | 0.8 | 0.00 | 0.11764706 |
|  | **'FAR13-14'** | 120.6 | 16.6 | 0.4 | 0.00 | 5.77647059 |
|  | **'FAR14-15'** | 127.6 | 5.0 | 0.8 | 0.00 | 2.68235294 |
|  | **'FAR15-16'** | 90.8 | 6.8 | 0.6 | 0.00 | 1.69411765 |
|  | **'FPR1-2'** | 111.6 | 5.0 | 1.4 | 1.00 | 0.01176471 |
|  | **'FPR2-3'** | 116.4 | 25.8 | 1.0 | 4.40 | 0.02352941 |
|  | **'FPR3-4'** | 77.6 | 26.6 | 1.2 | 5.00 | 0 |
|  | **'FPR4-5'** | 45.2 | 9.4 | 0.6 | 4.00 | 0.15294118 |
|  | **'FPR5-6'** | 41.2 | 5.0 | 0.8 | 2.00 | 0.35294118 |
|  | **'FPR6-7'** | 37.0 | 6.8 | 1.4 | 1.00 | 0.94117647 |
|  | **'FPR7-8'** | 57.8 | 3.2 | 0.4 | 0.60 | 0.88235294 |
|  | **'FPR9-10'** | 73.4 | 24.2 | 2.0 | 5.40 | 0 |
|  | **'FPR10-11'** | 81.8 | 27.0 | 2.8 | 7.00 | 0 |
|  | **'FPR11-12'** | 66.0 | 36.4 | 0.8 | 8.00 | 0.04705882 |
|  | **'FPR12-13'** | 67.4 | 33.8 | 1.0 | 8.40 | 0.44705882 |
|  | **'FPR13-14'** | 80.8 | 12.6 | 1.2 | 6.80 | 1.07058824 |
|  | **'FPR14-15'** | 65.0 | 19.8 | 1.4 | 7.00 | 0.03529412 |
|  | **'FPR15-16'** | 81.6 | 19.8 | 0.6 | 5.20 | 0.02352941 |
|  | **'TR1-2'** | 46.6 | 9.6 | 1.6 | 1.40 | 9.8 |
|  | **'TR2-3'** | 48.6 | 9.8 | 1.2 | 5.20 | 6.07058824 |
|  | **'TR3-4'** | 38.0 | 17.4 | 2.0 | 6.00 | 4.17647059 |
|  | **'TR4-5'** | 32.6 | 13.6 | 1.8 | 5.80 | 1.52941176 |
|  | **'TR5-6'** | 31.0 | 3.6 | 0.2 | 2.20 | 0.11764706 |
|  | **'TR6-7'** | 34.4 | 5.4 | 0.8 | 0.00 | 0 |
|  | **'TR7-8'** | 30.2 | 3.8 | 0.4 | 0.00 | 0 |
|  | **'TR8-9'** | 18.4 | 2.2 | 0.0 | 0.00 | 0 |
|  | **'TR9-10'** | 3.4 | 2.4 | 0.0 | 0.00 | 0 |
| Threshold |  |  |  |  | 7.65 | 5.9676 |

| **Patient 18** | **Channels** | **Ripples** | **FR** | **FRandR** | **Spikes** | **S + HFO** |
| --- | --- | --- | --- | --- | --- | --- |
| **ILAE 5** | **'IHAL1-2'** | 31.3 | 2.8 | 0.2 | 0.000 | 0.0 |
|  | **'IHAL2-3'** | 41.4 | 2.4 | 0.0 | 0.040 | 0.0 |
|  | **'IHAL3-4'** | 80.4 | 2.3 | 0.6 | 0.080 | 0.0 |
|  | **'IHPL1-2'** | 162.8 | 7.7 | 4.4 | 0.040 | 0.0 |
|  | **'IHPL2-3'** | 64.2 | 2.5 | 0.4 | 0.040 | 0.0 |
|  | **'IHPL3-4'** | 16.8 | 2.2 | 0.1 | 0.240 | 0.0 |
|  | **'PLL1-2'** | 139.0 | 20.2 | 5.9 | 0.000 | 0.0 |
|  | **'PLL2-3'** | 137.3 | 14.1 | 7.4 | 0.000 | 0.0 |
|  | **'PLL3-4'** | 86.6 | 3.3 | 1.3 | 0.000 | 0.0 |
|  | **'PLL4-5'** | 65.2 | 4.2 | 1.0 | 0.000 | 0.0 |
|  | **'PLL5-6'** | 43.0 | 2.6 | 0.4 | 0.200 | 0.0 |
|  | PM1-2 |  |  |  | 0.000 | 0.0 |
|  | PM2-3 |  |  |  | 0.000 | 0.0 |
|  | **'PML3-4'** | 18.8 | 2.6 | 0.1 | 0.000 | 0.0 |
|  | **'PML4-5'** | 34.6 | 2.1 | 0.2 | 0.000 | 0.0 |
|  | **'PML5-6'** | 41.1 | 1.8 | 0.3 | 0.200 | 0.0 |
|  | **'TL1-2'** | 0.2 | 2.2 | 0.0 | 0.000 | 0.0 |
|  | **'TL2-3'** | 1.1 | 4.1 | 0.6 | 0.000 | 0.0 |
|  | **'TL3-4'** | 8.3 | 29.4 | 1.8 | 0.000 | 0.0 |
|  | **'TL4-5'** | 21.4 | 25.88 | 1.4 | 0.000 | 0.0 |
|  | **'TL5-6'** | 19.2 | 4.1 | 0.0 | 0.200 | 0.0 |
|  | **'TL6-7'** | 11.5 | 4.1 | 0.1 | 0.000 | 0.0 |
|  | **'TL7-8'** | 1.5 | 2.6 | 0.0 | 0.000 | 0.0 |
|  | **'TL8-9'** | 3.8 | 2.6 | 0.0 | 0.000 | 0.0 |
|  | **'TL9-10'** | 2.1 | 2.8 | 0.0 | 0.000 | 0.0 |
| Threshold |  |  |  |  | 0.21 |  |

| **Patient 19** | **Channels** | **Ripples** | **FR** | **FRandR** | **Spikes** | **S + HFO** |
| --- | --- | --- | --- | --- | --- | --- |
| **ILAE 6** | **PL1-2'** |  |  |  | 0 | 0 |
|  | **'PL2-3'** | 34.1 | 1.9 | 0.4 | 0 | 0 |
|  | **'PL3-4'** | 32.8 | 1.8 | 0.1 | 0 | 0 |
|  | **'PL4-5'** | 55.0 | 2.8 | 0.8 | 0 | 0 |
|  | **'PL5-6'** | 55.8 | 3.7 | 0.8 | 0.61538462 | 0.0769 |
|  | **'PL6-7'** | 35.5 | 3.5 | 0.4 | 0.67692308 | 0.0462 |
|  | **'PL7-8'** | 24.3 | 3.3 | 0.4 | 0.4 | 0.0308 |
|  | **PL9-10'** |  |  |  | 0 | 0 |
|  | **'PL10-11'** | 38.9 | 2.6 | 0.4 | 0 | 0 |
|  | **'PL11-12'** | 32.2 | 2.7 | 0.5 | 0 | 0 |
|  | **'PL12-13'** | 55.9 | 4.1 | 1.4 | 0 | 0 |
|  | **'PL13-14'** | 71.3 | 22.6 | 3.6 | 5.69230769 | 0.2 |
|  | **'PL14-15'** | 64.3 | 21.5 | 3.0 | 6.2 | 0.2 |
|  | **'PL15-16'** | 55.2 | 4.6 | 1.4 | 1.6 | 0.1538 |
|  | **PL17-18'** |  |  |  | 0 | 0 |
|  | **'PL18-19'** | 44.9 | 2.8 | 0.6 | 0 | 0 |
|  | **'PL19-20'** | 29.5 | 2.3 | 0.4 | 0 | 0 |
|  | **'PL20-21'** | 7.4 | 2.5 | 0.0 | 0.01538462 | 0 |
|  | **'PL21-22'** | 16.1 | 3.6 | 0.6 | 0.06153846 | 0.0308 |
|  | **'PL22-23'** | 38.8 | 19.4 | 11.9 | 0.13846154 | 0.0769 |
|  | **'PL23-24'** | 32.9 | 15.7 | 10.2 | 0.21538462 | 0.0923 |
|  | **PL25-26'** |  |  |  | 0 | 0 |
|  | **'PL26-27'** | 45.3 | 2.5 | 0.3 | 0 | 0 |
|  | **'PL27-28'** | 21.6 | 2.5 | 0.1 | 0 | 0 |
|  | **'PL28-29'** | 31.0 | 3.1 | 0.8 | 0.15384615 | 0.0154 |
|  | **'PL29-30'** | 32.1 | 7.1 | 0.6 | 0.35384615 | 0.0154 |
|  | **'PL30-31'** | 49.8 | 18.8 | 4.7 | 0.58461538 | 0.0923 |
|  | **'PL31-32'** | 42.8 | 16.5 | 8.2 | 0.50769231 | 0.2154 |
|  | **'TL1-2'** | 46.9 | 8.6 | 1.2 | 1.10769231 | 0.2 |
|  | **'TL2-3'** | 37.6 | 9.2 | 1.2 | 1.26153846 | 0.1846 |
|  | **'TL3-4'** | 62.7 | 7.6 | 0.6 | 1.10769231 | 0.0462 |
|  | **'TL4-5'** | 109.3 | 8.6 | 0.9 | 1.58461538 | 0.0154 |
|  | **'TL5-6'** | 92.8 | 6.0 | 0.9 | 1.56923077 | 0.1077 |
|  | **'TL6-7'** | 63.3 | 1.2 | 0.1 | 0.95384615 | 0.0154 |
|  | **'TL7-8'** | 42.2 | 3.3 | 0.5 | 0.09230769 | 0 |
|  | **'TL9-10'** | 39.2 | 10.0 | 1.3 | 0.53846154 | 0.0154 |
|  | **'TL10-11'** | 69.5 | 8.4 | 0.9 | 0.73846154 | 0.0769 |
|  | **'TL11-12'** | 60.6 | 4.8 | 0.6 | 0.75384615 | 0.0154 |
|  | **'TL12-13'** | 28.0 | 3.2 | 0.7 | 0.78461538 | 0.1846 |
|  | **'TL13-14'** | 59.6 | 3.6 | 0.7 | 0.78461538 | 0.1231 |
|  | **'TL14-15'** | 66.9 | 2.8 | 0.6 | 0.69230769 | 0.1231 |
|  | **'TL15-16'** | 71.3 | 2.4 | 0.5 | 0.44615385 | 0 |
| Threshold |  |  |  |  | 3.2369 | 0.2000 |

| **Patient 20** | **Channels** | **Ripples** | **FR** | **FRandR** | **Spikes** | **S + HFO** |
| --- | --- | --- | --- | --- | --- | --- |
| **ILAE 5** | OTL1-2' |  |  |  | 0.0207 | 0 |
|  | **'OTL2-3'** | 98.0 | 3.7 | 1.2 | 0 | 0 |
|  | **'OTL3-4'** | 105.0 | 3.5 | 1.0 | 0 | 0 |
|  | **'OTL4-5'** | 75.8 | 3.2 | 0.6 | 0 | 0 |
|  | **'OTL5-6'** | 13.6 | 2.9 | 0.4 | 0.6069 | 0.0897 |
|  | **'OTL6-7'** | 56.8 | 4.4 | 0.9 | 1.8069 | 0.1586 |
|  | **'OTL7-8'** | 95.4 | 5.7 | 1.5 | 1.7793 | 0.1655 |
|  | **'OTL9-10'** | 59.8 | 6.3 | 1.3 | 3.9793 | 0.4759 |
|  | **'OTL10-11'** | 56.2 | 8.6 | 1.6 | 6.2 | 0.7034 |
|  | **'OTL11-12'** | 85.9 | 7.2 | 1.2 | 5.3793 | 0.4414 |
|  | **'OTL12-13'** | 96.3 | 3.5 | 0.9 | 0.069 | 0 |
|  | **'OTL13-14'** | 65.4 | 2.7 | 0.5 | 0.1931 | 0.0276 |
|  | **'OTL14-15'** | 15.8 | 3.0 | 0.4 | 0.1862 | 0.0483 |
|  | **'OTL15-16'** | 57.4 | 2.8 | 0.8 | 0.0621 | 0 |
| Threshold |  |  |  |  | 6.0359 | 0.6579 |

**TABLE 1S. Detailed resected area (red) , HFO, Spike and Spike+HFO rates and area (green) in each patient.**

| **HFO area** | Seizure recurrence | Seizure freedom |
| --- | --- | --- |
| not fully resected | 4 | 0 |
| fully resected | 3 | 13 |

| **Spikes area** | Seizure recurrence | Seizure freedom |
| --- | --- | --- |
| not fully resected | 5 | 6 |
| fully resected | 2 | 7 |

| **Spikes + HFO area** | Seizure recurrence | Seizure freedom |
| --- | --- | --- |
| not fully resected | 4 | 2 |
| fully resected | 2 | 11 |

**TABLE 2S. Confusion matrices for HFO area, Spikes area and Spikes+HFO area, according to the scheme proposed in Figure 2.**
